# A Three-Branch Time-Frequency Feature Fusion Method Based on Terahertz Signals for Identifying Delamination Defects in Composite Materials

**DOI:** 10.3390/s26082429

**Published:** 2026-04-15

**Authors:** Shengkai Yan, Jianguo Gao, Qiang Wang, Qiuhan Liu, Jiayang Yu, Jiajin Li, Gaocheng Chen

**Affiliations:** Equipment Management and Unmanned Aerial Vehicle Engineering School, Air Force Engineering University, Xi’an 710051, China; yanshengkai2002@163.com (S.Y.); weskr@foxmail.com (Q.W.); ayden.liu@foxmail.com (Q.L.); y1312887589@163.com (J.Y.); jennifer029998@163.com (J.L.); colingchy@163.com (G.C.)

**Keywords:** terahertz nondestructive testing, composite materials, delamination defects, time-frequency feature fusion, deep learning

## Abstract

Composite materials are critical components in advanced equipment such as aerospace, however, delamination defects that readily arise during manufacturing and in service present serious risks to equipment safety. Terahertz non-destructive testing is highly effective for analyzing the internal structure of composite materials, making it an effective approach for precise identification of delamination defects. Current terahertz detection approaches mainly depend on single domain features, making it difficult to capture complementary information from both the time and frequency domains. To address this, a Time-Frequency Feature-fusion Network (TFFN) is proposed. In this network, a three-branch architecture is employed: local transient patterns and pulse-related structural features are extracted by the local time-frequency branch; damage-sensitive frequency bands are focused on by the frequency-domain branch through a channel-space-frequency band attention mechanism; and deep integration of time-frequency features is achieved by the time-frequency fusion branch using Manifold Mixup. Finally, the features extracted from the three branches are adaptively fused via a cross-branch attention mechanism, and defect identification is accomplished by the classifier. Experimental results show that this method achieves accuracies of 98.40% on the glass fiber reinforced polymer (GFRP) dataset and 98.63% on the quartz fiber reinforced polymer (QFRP) dataset, surpassing the best existing method by 2% and 1.25%, respectively. A substantial improvement in both defect identification accuracy and the model’s generalization ability for layered structures is thereby achieved.

## 1. Introduction

Composite materials, as key foundational components in contemporary industrial systems, have become increasingly essential in diverse sectors such as aerospace, transportation, and civil engineering due to their outstanding characteristics, including high strength, low weight, corrosion resistance, and great design flexibility [1,2,3]. However, during both the manufacturing and service life of composites, defects such as delamination, porosity, and cracking are likely to occur, which can severely reduce their performance and durability. Among these, delamination defects, which are often hidden within the internal structure and evade detection by visual inspection, pose a particularly serious risk to the long-term reliability of composite structures [4,5]. Consequently, accurately identifying delamination is of great engineering importance for ensuring structural safety and the dependable operation of equipment.

Currently, multiple non-destructive [6,7,8] testing techniques are used to identify delamination defects in composite materials, such as ultrasonic inspection, infrared thermography, and microwave testing. Each of these methods, however, has notable limitations in delamination evaluation: ultrasonic testing depends on coupling media and faces a trade-off between penetration depth and image resolution [9,10]; infrared thermography requires materials with favorable thermal properties and is generally restricted to detecting defects near the surface [11,12,13]; microwave testing has low sensitivity to small-scale defects, and its signals are easily influenced by changes in the material’s dielectric properties [14,15]. In recent years, terahertz time-domain spectroscopy (THZ-TDS) has become a focus of research and application in composite Non-Destructive Testing (NDT) owing to its distinct advantages [16,17]. Relative to the above techniques, THZ-TDS offers a non-contact, non-ionizing inspection approach. Terahertz radiation can readily pass through non-metallic, non-polar composites, such as GFRP, delivering high-resolution imaging and accurate characterization of internal, hidden defects. Moreover, recent studies in terahertz metamaterials and resonant structures have shown that engineered frequency-selective responses can significantly enhance the sensitivity to subtle dielectric or structural variations in composite media. For example, ultra-highly sensitive THz sensors based on electromagnetically induced transparency (EIT)-like effects have demonstrated strong resonance confinement and high-Q spectral responses, enabling precise detection of small refractive-index perturbations [18]. Related dual-band EIT terahertz metasurface designs also exhibit strong field localization and enhanced interaction with layered media, providing valuable insights for improving damage-sensitive feature extraction in THz NDT scenarios [19]. These works indicate that the THz frequency domain carries rich discriminative cues and should be leveraged more effectively in defect characterization. In addition to these EIT-based THz sensing structures, recent progress in all-dielectric metasurfaces has further highlighted the critical role of high-Q resonant responses in frequency-domain detection. All-dielectric platforms supporting Fano and quasi-bound-state-in-the-continuum (quasi-BIC) resonances can produce extremely sharp spectral features and strong near-field enhancement, enabling highly sensitive refractive-index and molecular detection across multiple scenarios [20]. These studies collectively indicate that frequency-selective resonances encode rich, defect-sensitive information and provide compelling evidence that the frequency domain should be fully utilized in THz-based nondestructive evaluation.

Given these properties, THz technology has been extensively applied to detect delamination in composite materials [21,22]. To overcome the difficulty of thoroughly identifying and characterizing concealed multi-layer delamination in GFRP laminates, a THz-TDS imaging system was designed by Chung-Hyeon Ryu et al. [23]. Efficient detection and imaging of the delamination’s shape, thickness, and depth location were successfully achieved by examining how terahertz pulses interact with the material. Shi et al. [16] tackled the problem of precisely identifying internal defects during the production of GFRP. A technique that integrates cross-correlation-based extraction of pulse response functions from terahertz time-domain spectroscopy with image enhancement was proposed. The localization and imaging of deep and minute defects were achieved via this method, while the reliability and precision of non-destructive testing for composites were enhanced. To tackle the problem of automatically characterizing delamination in curved QFRP, a terahertz time-domain signal classification method based on Transformer neural networks was introduced by Liu et al. [24]. By combining this method with a collaborative robot, an automated detection system was established that enables high-precision automatic recognition and visual positioning of pre-embedded delamination defects. Mu Da et al. [25] investigated the problem of decreased contrast and reduced accuracy in defect characterization during terahertz imaging of internal flaws in ultra-multilayer glass fiber-reinforced polymer composites, which arise from signal attenuation and multiple reflections. They proposed a terahertz imaging characterization framework based on reconstructing the lamination structure and performing pixel-level clustering, utilizing the local symmetry of reflected pulses and the density of point clouds. Xu et al. [26] tackled the problem of performance degradation in data-driven THZ non-destructive evaluation under changing testing conditions, which frequently causes shifts in data distributions and weakens model generalization. An intelligent THz 3D characterization framework that leverages a deep adversarial domain adaptation strategy was proposed. Through the use of unsupervised adversarial learning, discrepancies between various THz datasets were substantially reduced, enabling highly accurate automated localization and imaging of delamination defects in composite materials operating under complex conditions.

At the same time, the computer-vision community has demonstrated that adaptive feature enhancement can substantially improve robustness under challenging sensing conditions. For instance, adaptive learning filters embedded in Vision Transformers (ALF-ViT) have been shown to significantly enhance pixel-level segmentation quality in low-light or noisy environments by dynamically amplifying informative regions while suppressing background interference [27]. These findings highlight the importance of adaptive, noise-aware, and domain-enhanced representation learning—capabilities that are likewise crucial for THz-TDS signals affected by attenuation, scattering, and multilayer reflections. The aforementioned studies have achieved certain advances, but their core approach still focuses on a single category of features. Whether improving algorithms using time-domain impulse responses or employing deep learning to classify time-domain waveforms or images, these approaches essentially extract features exclusively from the time-domain perspective. Such a one-dimensional feature representation leads to reduced accuracy and robustness when facing complex detection conditions, especially in the presence of complicated attenuation effects and noise interference [28,29,30]. To tackle the aforementioned problems, several researchers have sought to improve detection accuracy through multi-domain feature fusion techniques. Tu et al. [31] developed a multi-damage identification method based on multi-domain feature fusion combined with machine learning for terahertz non-destructive testing of defective epoxy coating structures. By extracting features from the time domain, frequency domain, and wavelet packet energy, employing an improved random forest algorithm for feature selection, and adopting a cascaded support vector machine classifier tuned through particle swarm optimization, defects of various types and severities were accurately classified and evaluated. The challenge of automatic and reliable detection of interfaces and defects in coating-to-bonding structures during terahertz signal analysis was addressed by Cui et al. [32], and a framework integrating Residual Networks with Bidirectional Long Short-Term Memory networks was proposed. By feeding the signals into a spatio-temporal feature extraction module, their method enabled precise automatic localization of coating interfaces thicker than 80 µm, as well as high-accuracy detection of defects within the bonding layer. Although some studies have sought to integrate multi-domain features, existing methods still suffer from several limitations. On the one hand, multi-domain features are typically combined through simple concatenation, without mechanisms for deep interaction or adaptive fusion among the features [33,34]. On the other hand, the feature extraction process is still largely restricted to the time domain or the spatiotemporal domain and does not sufficiently leverage multi-scale information in the frequency and time–frequency domains.

TFFN for the classification of delamination defects in composite materials is proposed. A feature fusion framework integrating local time-frequency, frequency domain, and time–frequency fused representations is constructed to achieve deep and complementary integration of multi-domain information. This approach improves both the accuracy and robustness of delamination defect identification in composite materials. The main contributions are summarized as follows:

1. A three-branch feature fusion framework is proposed. Features extracted from the local time-frequency, frequency domain, and time–frequency domain branches are combined, thereby addressing the limitations of single-view representations. This architecture provides a comprehensive basis for multi-domain information extraction and deep feature integration from terahertz signals.
2. A frequency-domain adaptive enhancement module, named the channel–spatial–frequency attention network (CSFANet), is introduced. This module performs adaptive decomposition in the frequency domain using deformable convolutions, strengthens feature interactions through a unified attention mechanism across frequency, spatial, and channel dimensions, and applies an adaptive weighting scheme to highlight critical information while attenuating noise.
3. Two feature fusion strategies are introduced. First, the Manifold Mixup method is creatively utilized in the time-frequency branch, where linear interpolation between time domain and frequency domain features is carried out in the feature space to achieve deep semantic alignment of time frequency representations, thereby boosting the robustness of defect classification. Second, a cross-branch attention module is developed to adaptively perform weighted fusion of the three-branch features, which facilitates the extraction of more discriminative fused features and further enhances classification accuracy.

## 2. A Time-Frequency Feature Fusion Based Composite Material Delamination Defect Classification Network

**Figure 1:**
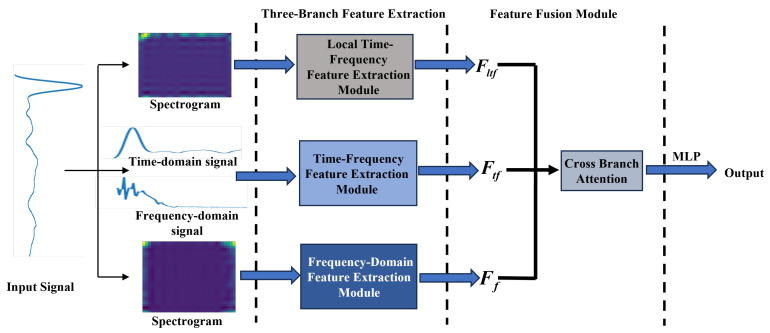
TFFN architecture. Colors indicate different feature domains: gray for local time–frequency, light blue for time–frequency, and dark blue for frequency.

To fully extract multi-domain information from terahertz signals, TFFN is proposed in this paper, as shown in Figure 1. The local time-frequency branch is designed to capture localized transient patterns and pulse-related structures from the time-frequency spectrogram. In the frequency-domain branch, a three-dimensional channel–spatial–frequency attention mechanism is employed to adaptively highlight frequency ranges that are sensitive to damage. The Manifold Mixup strategy is utilized in the time-frequency fusion branch to achieve deep integration and semantic alignment of features from both the time and frequency domains. Regarding the detailed workflow, the original terahertz signals and their corresponding time–frequency spectrograms are first input into their respective three branches. Features from the local time-frequency, frequency-domain, and temporal-frequency fusion branches are extracted independently and subsequently fused to obtain comprehensive multi-domain representations. Finally, a classification network is employed to identify and categorize the defects.

### 2.1. Loacl Time-Frequency Feature Extraction

The local time-frequency branch is designed to extract the distinctive manifestations of local dynamics evolving over time in the time-frequency domain. Many defect-related patterns, such as short-term energy fluctuations, transient responses, and local vibration modes, essentially manifest as time-varying local dynamics; however, these are often difficult to observe directly in raw time-domain waveforms, whereas they form a clearer local energy structure in the time-frequency representation.

**Figure 2:**
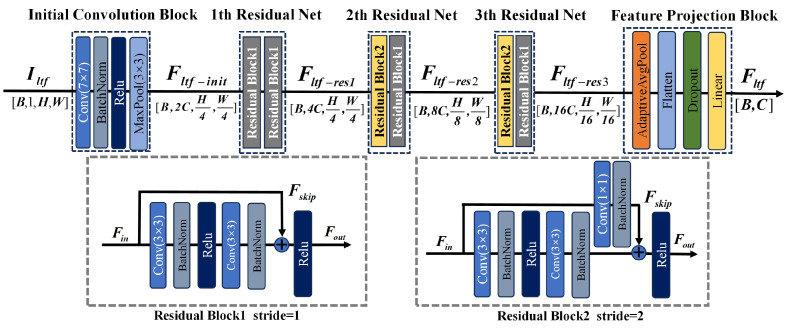
Local time-frequency branch architecture.

Time–frequency spectrograms are used as inputs to the time-domain feature extraction branch. One-dimensional time-domain signals are transformed into these spectrograms using the Short-Time Fourier Transform. By this transformation, the information dimension is expanded, feature discriminability is improved, and a richer information basis is provided for extracting features from non-stationary signals. Although the time-frequency spectrum expands the information dimension, it still exhibits stronger regularities compared with images in general computer vision tasks during defect detection. Therefore, a simplified ResNet architecture is used in this section to extract localized time-frequency features. By reducing redundant convolutional layers and the number of channels, the model complexity is significantly lowered while ensuring the ability to extract key time-frequency patterns patterns. The network structure is shown in Figure 2.

This branch is composed of an initial convolutional module, followed by three residual blocks and a feature projection module. The input time-frequency spectrogram $I_{ltf}\in R^{B\;\times \;1\;\times \;H\;\times \;W}$ is first fed into the initial convolutional layer, producing the initial feature representation $F_{lt\mathrm{f-init}}\in R^{B\;\times \;2C\;\times \;\frac{H}{4}\;\times \;\frac{W}{4}}$:

(1) $$F_{lt\mathrm{f-init}}=MaxPool_{3\;\times \;3,\;s=2}\left(ReLU\left(BN\left(Conv_{7\;\times \;7,\;s=2}\left(I_{ltf}\right)\right)\right)\right)$$

where this operation halves the spatial resolution while doubling the channel dimension to 2C. After the initial convolution, a max-pooling layer is applied to further compress the spatial information, thereby expanding the network’s receptive field and enhancing its robustness.

To capture detailed characteristics of the signal, a three-layer residual network is employed in the local time-frequency branch to extract multi-scale features: the first layer focuses on local spectro-temporal patterns, the second layer captures medium-range correlations, and the third layer learns broader-scale representations. The structure of the residual layer is illustrated in Figure 2, and the residual block is designed as follows:

(2) $$F_{out}=ReLU\left(BN\left(Conv_{3\;\times \;3,\;s}\left(ReLU\left(BN\left(Conv_{3\;\times \;3,\;s=2}\left(F_{in}\right)\right)\right)\right)\right)\right)+F_{skip}$$

where $F_{in}$ and $F_{out}$ are the inputs and outputs of the residual block, respectively, and $F_{skip}$ is the skip-connection vector. To maintain consistency in feature dimensions between the main pathway and the skip connection, a specific connection strategy is designed. If the input and output channel numbers match and the stride is 1, an identity mapping is adopted, allowing the input features to be passed through unchanged. Otherwise, a 1 × 1 convolution followed by batch normalization is used to transform the feature dimensions, thereby enabling valid residual addition. The formulation of $F_{skip}$ is defined as follows:

(3) $$F_{skip}=\left\{\begin{array}{cc} F_{in}, & C_{in}=C_{out}\;\mathrm{and}\;s=1\\ \mathrm{BN}\left({\mathrm{Conv}}_{1\;\times \;1,\;s,\;p}\left(F_{in}\right)\right), & \mathrm{otherwise} \end{array}\right.$$

where *s* is the stride of the convolutional layer. After feature extraction, the loacl time-frequency feature $F_{ltf}\in R^{C}$ is obtained through a feature projection module.

The feature projection module is made up of an adaptive average pooling layer, a flatten operation, a dropout layer, and a fully connected layer. It serves to compress high-dimensional spatiotemporal features into a concise global vector representation, while the introduced randomness through regularization helps improve the model’s generalization performance. The details are as follows:

(4) $$F_{ltf}=W\cdot Dropout\left(vec\left(GAP\left(F_{ltf-res3}\right)\right)\right)+b$$

where $W_{ltf}\in R^{B\;\times \;16C}$ and $b\in R^{C}$ are learnable parameters, vec is the flatten operation, and GAP is global average pooling.

### 2.2. Frequency-Domain Feature Extraction

**Figure 3:**
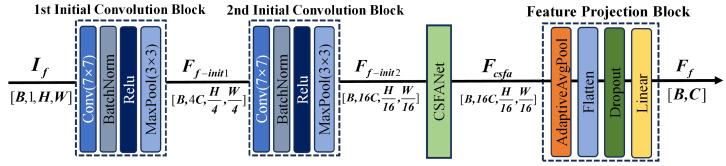
Frequency-domain branch network structure.

To capture the energy distribution and key spectral components of signals in the frequency domain, a frequency domain feature extraction branch that takes the time-frequency map as input is designed. The time-frequency spectrogram contains multi-scale frequency response information induced by defects. However, features across different frequency bands are often coupled during standard convolution operations, making it difficult for the network to focus on the critical frequency bands that are most discriminative for classification. To tackle this problem, CSFANet is introduced in the frequency domain feature extraction branch. In this model, the mixed features are first decomposed into sub-features via dynamic frequency band partitioning within CSFANet. Then, the significance of these sub-features is assessed and adaptively reweighted through a combined channel-spatial-frequency attention mechanism, thereby improving the discriminative capability of the mixed features. The network structure of the frequency-domain branch is illustrated in Figure 3.

As shown in Figure 3, the frequency-domain branch is composed of two initial convolutional modules, a frequency-domain adaptive enhancement module, and a feature projection module. The input signal $I_{f}\in R^{B\;\times \;1\;\times \;H\;\times \;W}$ is first processed by two consecutive convolutional layers, yielding $F_{\mathrm{f-init}1}\in R^{B\;\times \;4C\;\times \;\frac{H}{4}\;\times \;\frac{W}{4}}$ and $F_{\mathrm{f-init}2}\in R^{B\;\times \;16C\;\times \;\frac{H}{16}\;\times \;\frac{W}{16}}$, respectively. These feature maps are subsequently fed into the frequency-domain adaptive enhancement module to obtain the enhanced feature $F_{csfa}\in R^{B\;\times \;16C\;\times \;\frac{H}{16}\;\times \;\frac{W}{16}}$. Finally, the feature projection module converts this representation into the frequency-domain vector $F_{f}\in R^{B\;\times \;C}$.

**Figure 4:**
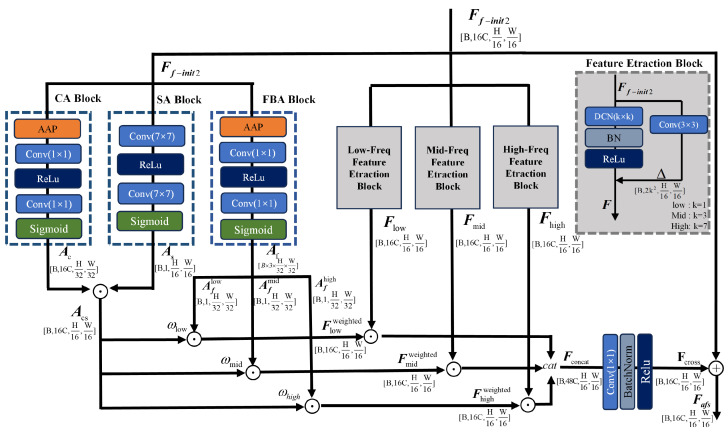
Architecture of the CSFANet.

CSFANet serves as the core component of the frequency-domain branch and is described in detail in this subsection. Its primary function is to adaptively extract and amplify the most discriminative frequency-domain information for the classification task along the frequency dimension, thereby strengthening frequency-domain representations and enhancing the model’s global perception of subtle structural details. The architecture of CSFANet is shown in Figure 4. Its input is $F_{\mathrm{f-init}2}\in R^{B\;\times \;16C\;\times \;\frac{H}{16}\;\times \;\frac{W}{16}}$, and it mainly consists of three parts: frequency-domain feature decomposition, three-dimensional joint attention, and feature weighting and fusion. The specific components are shown as below.

#### 2.2.1. Frequency-Domain Feature Decomposition

Different frequency ranges within an image encode distinct levels of semantic content. Low-frequency components are typically used to describe smooth regions and global shapes, mid-frequency components are employed to capture key textures and structural patterns, and high-frequency components are mainly associated with edges, noise, or subtle local variations. In this module, deformable convolutions are used to decompose features in the frequency domain, splitting the input into low-, mid-, and high-frequency bands. By learning and applying spatial offsets, the network achieves frequency-domain decomposition and generates separate feature representations corresponding to each frequency band.

The offset generation network is designed to learn the sampling offsets for the deformable convolutions. It is composed of three distinct 3 × 3 convolutional layers, which correspond to the low-, mid-, and high-frequency branches, respectively. Given the input feature map $F_{\mathrm{f-init}2}\in R^{B\;\times \;16C\;\times \;\frac{H}{16}\;\times \;\frac{W}{16}}$, the offsets output by the three branches are $\Delta_{\mathrm{low}}\in R^{B\;\times \;2k_{\mathrm{low}}^{2}\;\times \;\frac{H}{16}\;\times \;\frac{W}{16}}$, $\Delta_{\mathrm{mid}}\in R^{B\;\times \;2k_{\mathrm{mid}}^{2}\;\times \;\frac{H}{16}\;\times \;\frac{W}{16}}$, and $\Delta_{\mathrm{high}}\in R^{B\;\times \;2k_{\mathrm{high}}^{2}\;\times \;\frac{H}{16}\;\times \;\frac{W}{16}}$. These offsets determine the sampling positions of the convolution kernels, allowing them to focus on features across different frequency bands, where $2k^{2}$ is the number of offset parameters for each spatial location, with *k* denoting the kernel size, and the kernel sizes satisfy $k_{\mathrm{low}}>k_{\mathrm{mid}}>k_{\mathrm{high}}$. In our implementation, the specific values are set as $k_{\mathrm{low}}=7$, $k_{\mathrm{mid}}=3$, and $k_{\mathrm{high}}=1$.

After obtaining the offsets, three sets of deformable convolutions with different scales are applied to the input feature $F_{f\_init2}$ to perform dynamic frequency-band decomposition, yielding low-, mid-, and high-frequency sub-band features $F_{low},F_{mid},F_{high}\in R^{B\;\times \;16C\;\times \;\frac{H}{16}\;\times \;\frac{W}{16}}$:

(5) $$\begin{array}{cc} F_{low} & =ReLU\left(BN\left(DCN_{7\;\times \;7}\left(F_{\mathrm{f-init}2},\Delta_{low}\right)\right)\right) \end{array}$$

(6) $$\begin{array}{cc} F_{mid} & =ReLU\left(BN\left(DCN_{3\;\times \;3}\left(F_{\mathrm{f-init}2},\Delta_{mid}\right)\right)\right) \end{array}$$

(7) $$\begin{array}{cc} F_{high} & =ReLU\left(BN\left(DCN_{1\;\times \;1}\left(F_{\mathrm{f-init}2},\Delta_{high}\right)\right)\right) \end{array}$$

where global frequency-domain trends are captured by the low-frequency branch ($k_{low}=7$), local frequency-domain textures are extracted by the mid-frequency branch ($k_{mid}=3$), and detailed frequency-domain variations are focused on by the high-frequency branch ($k_{high}=1$), thereby forming complementary features across the full frequency band.

#### 2.2.2. Three-Dimensional Joint Attention

To address the limitations of conventional attention mechanisms, which usually focus on just one dimension, a three-dimensional joint attention mechanism is proposed in this section, integrating channel, spatial, and frequency information. This mechanism is composed of two parallel sub-modules: a channel–spatial joint attention module and a frequency-band attention module. The outputs of these two sub-modules are subsequently weighted and combined to generate an integrated attention map with multi-dimensional perception capability. For the input feature $F_{\mathrm{f-init}2}\in R^{B\;\times \;16C\;\times \;\frac{H}{16}\;\times \;\frac{W}{16}}$, the computational procedures of the two sub-modules are detailed below.

- Channel–Spatial Joint Attention (CSJA)

To capture inter-channel dependencies and spatial location dependencies in frequency-domain features, a Channel–Spatial Joint Attention (CSJA) module is designed. Channel-wise weights $A_{c}$ and spatial region weights $A_{s}$ are generated through parallel branches, respectively, and are then element-wise multiplied to achieve a synergistic enhancement of channel and spatial information.

Channel attention is employed to reassign importance to each channel. First, global pooling is applied to the input feature $F_{\mathrm{f-init}2}$. Subsequently, two convolutional layers are used for nonlinear transformation, and finally, a Sigmoid activation generates the channel-wise weight $A_{c}\in R^{B\;\times \;16C\;\times \;\frac{H}{32}\;\times \;\frac{W}{32}}$:

(8) $$A_{c}=Sigmoid\left(Conv_{1\;\times \;1}\left(ReLU\left(BN\left(Conv_{1\;\times \;1}\left(AAP\left(F_{\mathrm{f-init}2}\right)\right)\right)\right)\right)\right)$$

where AAP denotes Adaptive Average Pooling, which is used to aggregate spatial information from the input feature map $F_{\mathrm{f-init}2}$ into a compact channel-wise descriptor.

The importance of spatial regions is determined with high computational efficiency by the spatial attention branch. A spatial weight map is generated through a lightweight two-layer convolutional encoder. Based on the distribution characteristics of the weight map, the regions are adaptively divided into high-, medium-, and low-importance categories, and corresponding attention weights $A_{s}\in R^{B\;\times \;1\;\times \;\frac{H}{16}\;\times \;\frac{W}{16}}$ are assigned accordingly:

(9) $$A_{s}=Sigmoid\left(Conv_{7\;\times \;7}\left(ReLU\left(BN\left(Conv_{7\;\times \;7}\left(F_{\mathrm{f-init}2}\right)\right)\right)\right)\right)$$

where convolution with a large receptive field is employed to transform pixel-wise attention into region-level weighting, thereby preserving global structural awareness while significantly reducing computational cost. The channel weight $A_{c}$ is broadcast to the spatial dimensions of the spatial weight $A_{s}$, followed by element-wise multiplication to yield the channel-spatial joint weight $A_{cs}\in R^{B\;\times \;16C\;\times \;\frac{H}{16}\;\times \;\frac{W}{16}}$.

- Frequency-band attention

To adaptively adjust the relative importance among low-, mid-, and high-frequency band features, global average pooling is first applied to the input feature $F_{f\_init2}$ to obtain a frequency band descriptor vector. Two 1 × 1 convolutional layers are then employed to produce the frequency band attention $A_{f}\in R^{B\;\times \;3\;\times \;\frac{H}{32}\;\times \;\frac{W}{32}}$, where the output channel size of 3 corresponds to the three frequency bands (low, mid, and high), respectively:

(10) $$A_{f}=Sigmoid\left(Conv_{1\;\times \;1}\left(ReLU\left(BN\left(Conv_{1\;\times \;1}\left(AAP\left(F_{\mathrm{f-init}2}\right)\right)\right)\right)\right)\right)$$

where AAP denotes the Adaptive Average Pooling operation applied to $F_{\mathrm{f-init}2}$ to compress its spatial dimensions, generating the compact frequency band descriptor vector that serves as the input for subsequent attention weight learning across the three frequency bands.

- Channel–spatial–frequency three-dimensional joint attention

The frequency-band attention $A_{f}$ is evenly split into three parts along the channel dimension, yielding $A_{f}^{low}$, $A_{f}^{mid}$, and $A_{f}^{high}\in R^{B\;\times \;1\;\times \;\frac{H}{32}\;\times \;\frac{W}{32}}$, which correspond to the low, mid, and high-frequency-bands, respectively. Each frequency-band attention component $A_{f}^{band}$ is first multiplied element-wise with the channel-spatial joint weight $A_{cs}$, and then weighted by a learnable frequency-band coefficient $\omega_{band}$:

(11) $$\begin{array}{cc} A_{\mathrm{final}}^{low} & =\omega_{low}\;\times \;A_{cs}⊙A_{f}^{low} \end{array}$$

(12) $$\begin{array}{cc} A_{\mathrm{final}}^{mid} & =\omega_{mid}\;\times \;A_{cs}⊙A_{f}^{mid} \end{array}$$

(13) $$\begin{array}{cc} A_{\mathrm{final}}^{high} & =\omega_{high}\times A_{cs}⊙A_{f}^{high} \end{array}$$

where $\omega_{low},\omega_{mid},\omega_{high}\in R$ are learnable scalar weight parameters initialized to 1.0, 1.2, and 0.8, respectively. This initialization strategy is based on prior knowledge from frequency-domain analysis: a higher initial weight is assigned to mid-frequency components, as they generally contain major texture and structural information; a moderate weight is given to low-frequency components, which represent global trends; and a lower initial weight is set for high-frequency components since they may contain more details and noise. During training, these parameters are adaptively adjusted according to the task requirements, thereby learning an optimal configuration of frequency-band importance.

#### 2.2.3. Feature Weighting and Fusion

Based on the frequency-band features extracted in Section 2.2.1 and the integrated attention weights computed in Section 2.2.2, feature weighting is applied in this section to assign the attention weights to their corresponding frequency-band features, as formulated below:

(14) $$\begin{array}{cc} F_{\mathrm{low}}^{weighted} & =F_{\mathrm{low}}⊙A_{\mathrm{final}}^{\mathrm{low}} \end{array}$$

(15) $$\begin{array}{cc} F_{\mathrm{mid}}^{weighted} & =F_{\mathrm{mid}}⊙A_{\mathrm{final}}^{\mathrm{mid}} \end{array}$$

(16) $$\begin{array}{cc} F_{\mathrm{high}}^{weighted} & =F_{\mathrm{high}}⊙A_{\mathrm{final}}^{\mathrm{high}} \end{array}$$

where $F_{low}$, $F_{mid}$, and $F_{high}\in R^{B\;\times \;16C\;\times \;\frac{H}{16}\;\times \;\frac{W}{16}}$ denote the low-, mid-, and high-frequency band features output by the dynamic frequency band decomposition module, respectively. The symbol ⊙ represents element-wise multiplication.

Although the three weighted frequency-band features are adjusted by attention, effective cross-band information integration is still required. Therefore, a fusion strategy combined with a residual connection to obtain the final feature is designed in this module.

First, the three weighted band features are concatenated along the channel dimension to obtain $F_{\mathrm{concat}}\in R^{B\;\times \;48C\;\times \;\frac{H}{16}\;\times \;\frac{W}{16}}$:

(17) $$F_{\mathrm{concat}}=\mathrm{concat}\left(F_{\mathrm{low}}^{\mathrm{weighted}},\;F_{\mathrm{mid}}^{\mathrm{weighted}},\;F_{\mathrm{high}}^{\mathrm{weighted}}\right)$$

where the features extracted from the different frequency bands are concatenated along the channel axis, thereby enabling subsequent cross-band interactions. As a result, global information from the low-frequency band, structural information from the mid-frequency band, and edge-related details from the high-frequency band are fused into the combined feature $F_{\mathrm{concat}}$.

To achieve efficient deep fusion of cross-band information, a 1 × 1 convolutional layer is employed for adaptive fusion in the channel dimension:

(18) $$F_{\mathrm{cross}}=\mathrm{ReLU}\left(\mathrm{BN}\left({\mathrm{Conv}}_{1\;\times \;1}\left(F_{\mathrm{concat}}\right)\right)\right)\in R^{B\;\times \;16C\;\times \;\frac{H}{16}\;\times \;\frac{W}{16}}$$

where ${\mathrm{Conv}}_{1\;\times \;1}$ denotes a convolution operation with a kernel size of 1 × 1, which reduces the channel count from 48C to 16C; BN denotes the batch normalization layer.

To preserve the original feature information and alleviate gradient vanishing, a residual connection is introduced to add the fused feature $F_{\mathrm{cross}}$ to the module input feature $F_{\mathrm{f-init}2}$, yielding $F_{\mathrm{afs}}\in R^{B\;\times \;16C\;\times \;\frac{H}{16}\;\times \;\frac{W}{16}}$.

Finally, similar to the time-domain branch, adaptive average pooling, flattening, and a fully connected projection are sequentially applied to $F_{\mathrm{afs}}$ to obtain the frequency-domain feature $F_{f}$.

### 2.3. Time-Frequency Domain Fusion Feature Extraction

**Figure 5:**
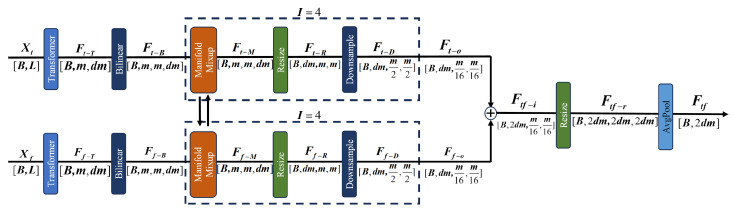
Time-frequency domain branch network.

To address the limitation that local time-frequency branches can only be modeled in a single time-frequency representation and cannot explicitly model the complementary relationships between the raw time-domain and frequency-domain representations, a time-frequency feature fusion module is proposed in this section to integrate transient physical information from the time domain with global structural features from the frequency domain, thereby capturing the cross-domain nonlinear relationship between the two. This module preserves precise temporal features of the signal while simultaneously capturing global frequency distribution patterns. In this module, the raw time-domain signal $X_{t}\in R^{B\;\times \;L}$ and the frequency-domain signal $X_{f}\in R^{B\;\times \;L}$ serve as inputs. The framework, illustrated in Figure 5, proceeds as follows: the time-domain and frequency-domain signals are input into Transformer encoders to capture long-range dependencies along the time dimension, extracting the respective time-domain and frequency-domain features. A bilinear transformation is then applied to combine them into a high-dimensional representation. Manifold Mixup is employed in the model space as a data augmentation technique to improve the model’s generalization ability. Finally, the features from both branches are fused to obtain the final time–frequency domain feature. The detailed procedure is outlined below.

#### 2.3.1. Transformer Encoding

**Figure 6:**
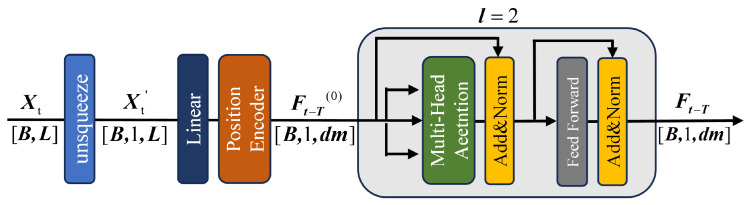
Transformer encoding network.

A Transformer-based encoding module is used for feature extraction. The time-domain and frequency-domain branches share the same network architecture. Using the time-domain branch as an illustration, its structure is shown in Figure 6. The input signal is initially passed through a projection layer and position encoding and is then input to the encoder, where multi-head attention and feed-forward networks are applied to derive the encoded feature representations.

The time-domain input signal $X_{t}$ and frequency-domain input signal $X_{f}$ are first reshaped to expand their dimensions, resulting in $X_{t}^{\prime }\in R^{B\;\times \;1\;\times \;L}$ and $X_{f}^{\prime }\in R^{B\;\times \;1\;\times \;L}$. A linear projection is then applied to transform the sequence length into the model’s hidden dimension $d_{m}$. To retain information about the order of the sequence, sinusoidal positional encodings are added, producing $F_{\mathrm{t-T}}^{\left(0\right)}\in R^{B\;\times \;1\;\times \;d_{m}}$ and $F_{\mathrm{f-T}}^{\left(0\right)}\in R^{B\;\times \;1\;\times \;d_{m}}$. These initial features $F_{\mathrm{t-T}}^{\left(0\right)}$ and $F_{\mathrm{f-T}}^{\left(0\right)}$ are then passed through Transformer encoders for further representation learning. Each encoder is composed of *l* stacked standard encoder blocks, and each block contains a multi-head self-attention module followed by a feed-forward network, both wrapped with residual connections and layer normalization. After propagating through all *l* layers, the time-domain encoded feature $F_{\mathrm{t-T}}^{\left(l\right)}$ and the encoded frequency-domain representation $F_{\mathrm{f-T}}^{\left(l\right)}$ are obtained.

#### 2.3.2. Bilinear Transformation

The bilinear transformation module is designed to capture second-order interaction information between the time-domain and frequency-domain encoded features. First, the encoded features are expanded in dimension: the time-domain encoded feature $E_{t}\in R^{B\;\times \;1\;\times \;d_{m}}$ and the frequency-domain encoded feature $E_{f}\in R^{B\;\times \;1\;\times \;d_{m}}$ are replicated *m* times along the sequence dimension, yielding the expanded features $E_{t}^{\prime }\in R^{B\;\times \;m\;\times \;d_{m}}$ and $E_{f}^{\prime }\in R^{B\;\times \;m\;\times \;d_{m}}$. A second-order feature representation is generated through outer- product operations:

(19) $$\begin{array}{cc} F_{\mathrm{t-B}} & =\frac{1}{\sqrt{d_{m}}}\left(E_{t}^{\prime }⊗E_{t}^{\prime }\right)\in R^{B\;\times \;m\;\times \;m\;\times \;d_{m}} \end{array}$$

(20) $$\begin{array}{cc} F_{\mathrm{f-B}} & =\frac{1}{\sqrt{d_{m}}}\left(E_{f}^{\prime }⊗E_{f}^{\prime }\right)\in R^{B\;\times \;m\;\times \;m\;\times \;d_{m}} \end{array}$$

where ⊗ denotes the outer product operation, which computes the element-wise product between features across different positions.

#### 2.3.3. Manifold Mixup

Given the challenges posed by mismatched feature distributions in bimodal signal fusion, simple methods like direct feature concatenation or basic weighted fusion are unable to capture deep semantic interactions and may lead to loss of information. To address this, Manifold Mixup is adopted for feature fusion in the time–frequency domain. Manifold Mixup is a data augmentation method operating in feature space. In this study, it is creatively utilized to derive fused time–frequency features, simultaneously achieving data augmentation and cross-modal fusion within the feature space. The total number of iterations is set to I=4, and the process for the *i*th iteration (i=1,…,I) is outlined as follows.

First, linear interpolation is carried out in the feature space between two randomly selected pairs of time-domain and frequency-domain features:

(21) $$\begin{array}{cc} F_{\mathrm{t-m}}^{\left(i\right)} & =\lambda_{i}F_{\mathrm{t-m}}^{\left(i-1\right)}+\left(1-\lambda_{i}\right)F_{\mathrm{f-m}}^{\left(i-1\right)} \end{array}$$

(22) $$\begin{array}{cc} F_{\mathrm{f-m}}^{\left(i\right)} & =\lambda_{i}F_{\mathrm{f-m}}^{\left(i-1\right)}+\left(1-\lambda_{i}\right)F_{\mathrm{t-m}}^{\left(i-1\right)} \end{array}$$

where $F_{\mathrm{t-m}}^{\left(0\right)}=F_{\mathrm{t-B}},\;F_{\mathrm{f-m}}^{\left(0\right)}=F_{\mathrm{f-B}}$ and $F_{\mathrm{t-m}}^{\left(K\right)}=F_{\mathrm{t-M}},\;F_{\mathrm{f-m}}^{\left(K\right)}=F_{\mathrm{f-M}}$. The mixing coefficient $\lambda_{i}∼U\left[0.4,0.6\right]$ follows a uniform distribution.

After each interpolation step, the feature dimensions are adjusted via a convolutional layer. Given the current feature tensors $F_{\mathrm{t-B}},F_{\mathrm{f-B}}\in R^{B\;\times \;m\;\times \;m\;\times \;d_{m}}$, a reshaping operation is performed to meet the format required by convolution, yielding $F_{\mathrm{t-R}}\in R^{B\;\times \;d_{m}\;\times \;m\;\times \;m}$ and $F_{\mathrm{f-R}}\in R^{B\;\times \;d_{m}\;\times \;m\;\times \;m}$. k × k convolutional layer is then applied for spatial downsampling:

(23) $$\begin{array}{cc} F_{\mathrm{t-D}} & ={\mathrm{Conv}}_{k\;\times \;k}^{\left(s\right)}\left(F_{\mathrm{t-R}}\right)\in R^{B\;\times \;d_{m}\;\times \;\frac{m}{2}\;\times \;\frac{m}{2}} \end{array}$$

(24) $$\begin{array}{cc} F_{\mathrm{f-D}} & ={\mathrm{Conv}}_{k\;\times \;k}^{\left(s\right)}\left(F_{\mathrm{f-R}}\right)\in R^{B\;\times \;d_{m}\;\times \;\frac{m}{2}\;\times \;\frac{m}{2}} \end{array}$$

where ${\mathrm{Conv}}_{k\;\times \;k}^{\left(s\right)}$ denotes a k × k convolution with stride *s*, performing both feature transformation and spatial down-sampling.

After *I* iterations, the final outputs are obtained as $F_{\mathrm{t-o}}\in R^{B\;\times \;d_{m}\;\times \;\frac{m}{16}\;\times \;\frac{m}{16}}$ and $F_{\mathrm{f-o}}\in R^{B\;\times \;d_{m}\;\times \;\frac{m}{16}\;\times \;\frac{m}{16}}$. The resulting time-frequency domain features $F_{\mathrm{t-o}}$ and $F_{\mathrm{f-o}}$ are concatenated to form $F_{\mathrm{tf-i}}\in R^{B\;\times \;2d_{m}\;\times \;\frac{m}{16}\;\times \;\frac{m}{16}}$. Subsequently, reshaping and pooling operations are performed to generate the final fused time–frequency domain feature $F_{tf}$.

This study introduces Manifold Mixup into the feature space. Unlike simple concatenation or additive fusion, which merely stack features without modeling cross-domain interactions, Manifold Mixup constructs smooth and continuous transitions between time-domain and frequency-domain features in the latent space. This is particularly suitable for terahertz signals, whose time- and frequency-domain representations originate from the same physical process and therefore exhibit natural semantic correspondence. In contrast, cross-attention-based feature alignment requires explicit query–key computations, bringing significant parameter overhead and posing a higher risk of overfitting under small-sample terahertz conditions. The interpolation-based alignment mechanism thus provides a lighter and more stable alternative.

### 2.4. Cross-Branch Attention Mechanism

**Figure 7:**
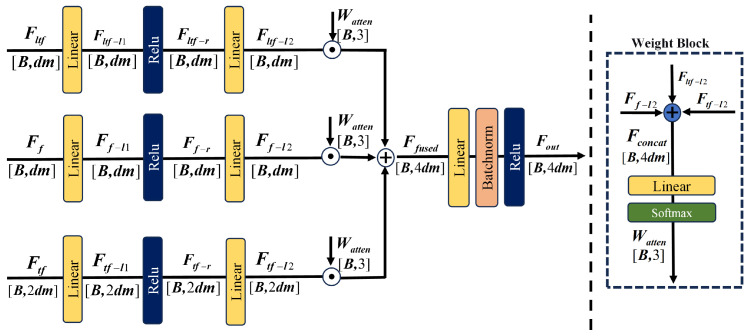
Cross-Branch attention network.

In this subsection, a feature fusion network based on cross-branch attention is proposed, as illustrated in Figure 7. The weight distributions of loacl time-frequency, frequency-domain, and time-frequency-domain branches are adaptively learned by the module, and complementary enhancement of multi-domain features is achieved through dynamic weighted fusion. The specific details are described below.

First, the features $F_{ltf}$, $F_{f}$, and $F_{tf}$ are individually processed by Linear, ReLU, and Linear layers, respectively, yielding the enhanced representations $F_{\mathrm{ltf-}12}\in R^{B\;\times \;d_{m}}$, $F_{\mathrm{f-}12}\in R^{B\;\times \;d_{m}}$, and $F_{\mathrm{tf-}12}\in R^{B\;\times \;d_{m}}$:

(25) $$\begin{array}{cc} F_{\mathrm{ltf-}l2} & =W_{2}^{\left(ltf\right)}\cdot \mathrm{ReLU}\left(W_{1}^{\left(ltf\right)}F_{f}+b_{1}^{\left(ltf\right)}\right)+b_{2}^{\left(ltf\right)} \end{array}$$

(26) $$\begin{array}{cc} F_{\mathrm{f-}l2} & =W_{2}^{\left(f\right)}\cdot \mathrm{ReLU}\left(W_{1}^{\left(f\right)}F_{f}+b_{1}^{\left(f\right)}\right)+b_{2}^{\left(f\right)} \end{array}$$

(27) $$\begin{array}{cc} F_{\mathrm{tf-}l2} & =W_{2}^{\left(tf\right)}\cdot \mathrm{ReLU}\left(W_{1}^{\left(tf\right)}F_{tf}+b_{1}^{\left(tf\right)}\right)+b_{2}^{\left(tf\right)} \end{array}$$

where $W_{2}^{\left(ltf\right)},W_{2}^{\left(f\right)},W_{2}^{\left(tf\right)}$ are learnable weight matrices and $b_{2}^{\left(ltf\right)},b_{2}^{\left(f\right)},b_{2}^{\left(tf\right)}$ are the corresponding bias terms.

The three enhanced features are concatenated along the feature dimension to obtain $F_{\mathrm{concat}}\in R^{B\;\times \;4d_{m}}$. Attention weights $W_{\mathrm{atm}}\in R^{B\;\times \;3}$ are then generated via a linear transformation followed by a Softmax function. Based on these attention weights, the three branch features are weighted and summed to produce the fused feature $F_{\mathrm{fused}}\in R^{B\;\times \;4d_{m}}$:

(28) $$F_{\mathrm{fused}}=W_{\mathrm{attn}}^{\left(1\right)}\cdot F_{\mathrm{ltf-}l2}+W_{\mathrm{attn}}^{\left(2\right)}\cdot F_{\mathrm{f-}l2}+W_{\mathrm{attn}}^{\left(3\right)}\cdot F_{\mathrm{tf-}l2}$$

where $W_{\mathrm{attn}}^{\left(1\right)}$, $W_{\mathrm{attn}}^{\left(2\right)}$, and $W_{\mathrm{attn}}^{\left(3\right)}$ correspond to the three components of $W_{\mathrm{attn}}$.

Finally, the output feature $F_{\mathrm{out}}\in R^{B\;\times \;4d_{m}}$ is obtained through a linear layer, batch normalization, and a ReLU activation function:

(29) $$F_{\mathrm{out}}=\mathrm{ReLU}\left(\mathrm{BN}\left(W_{3}\left(F_{\mathrm{fused}}\right)+b_{3}\right)\right)$$

where $W_{3}$ is a learnable weight matrix and $b_{3}$ is the corresponding bias term.

## 3. Experiment

### 3.1. Experimental Setup

**Figure 8:**
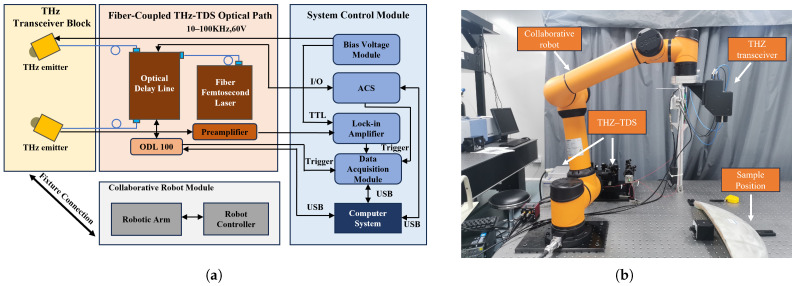
THZ detection system. (**a**) Schematic of the THz-TDS optical path. (**b**) Photograph of the detection system.

In this study, an automated detection method is implemented that combines an optical-fiber-coupled THz-TDS system with a collaborative robot. As shown in Figure 8, the robotic arm is employed as a motion carrier to drive the terahertz probe, enabling non-contact, adaptive, and nondestructive inspection of internal defects in composite materials. The core of the system is a self-developed optical-fiber-coupled THz-TDS instrument. Its excitation source is a fiber femtosecond laser with a pulse width of less than 90 fs, a repetition rate of 100 MHz, and a central wavelength of 1550 nm. A high-performance photoconductive antenna based on an InAlAs/InGaAs multilayer heterostructure is employed to generate and detect terahertz waves. The system exhibits excellent spectral performance: in fast and slow scanning modes, the spectral range reaches beyond 1.5 THz and 2.5 THz, respectively, with a spectral resolution better than 10 GHz and a dynamic range of 50–70 dB. An AUBO i5 six-axis collaborative robot (AUBO (Beijing) Robotics Technology Co., Ltd., Beijing, China) is employed in the experiments. It provides a working radius of 886.5 mm, with each joint offering a rotation range of ±175° and a positioning accuracy of ±0.02 mm. Scanning paths are programmed offline on a computer, allowing the robot to precisely control the terahertz transceiver module integrated at its end-effector, which enables automated inspection of complex curved surfaces. The SNR of the THz-TDS system, evaluated at the main pulse peak under the experimental configuration used in this work, is approximately 40 dB for both GFRP and QFRP specimens.

**Figure 9:**
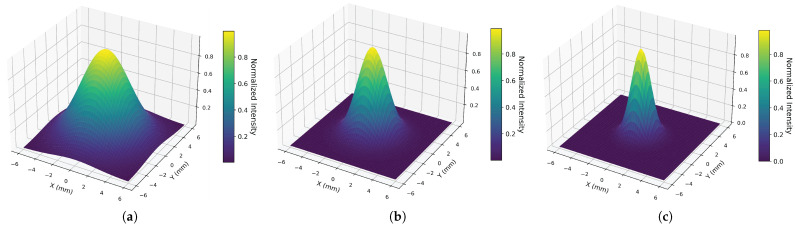
Three-dimensional THz beam profiles at different frequencies. (**a**) 0.3 THz, (**b**) 1.0 THz, (**c**) 2.0 THz.

To further verify the effective irradiation range of the terahertz probe during the scanning process, a three-dimensional spatial energy distribution map of the terahertz beam on the sample surface, based on beam waist parameters at different frequencies, is plotted as shown in Figure 9. It can be observed that the terahertz beam exhibits a typical quasi-Gaussian distribution on the sample surface, with the highest energy at the center of the spot and a rapid outward decay. As the frequency increases, the beam’s focusing capability improves, and the spot diameter gradually decreases: at 0.3 THz, 1.0 THz, and 2.0 THz, the beam waist radii are approximately 2.5 mm, 1.5 mm, and 1.0 mm, respectively.

The scanning step size used in this study is 0.5 mm, which is significantly smaller than the spot diameter under the aforementioned frequency conditions. Consequently, during the scanning process, the beam illumination areas of any adjacent sampling points overlapped extensively, ensuring continuous and thorough coverage of the entire detection area without any spatial gaps. These spot characteristics confirm that the experimental system exhibits excellent stability in terms of spatial coverage and provide a reliable energy basis for subsequent time-frequency domain feature analysis.

### 3.2. Specimen Preparation and Data Collection

#### 3.2.1. Design of Experimental Specimens

To validate the effectiveness of the proposed method, two types of composite sandwich specimens with predefined internal defects were designed and fabricated, made of GFRP and QFRP, respectively. Polytetrafluoroethylene (PTFE) films of specific dimensions were embedded between different ply layers to simulate delamination defects, enabling a systematic evaluation of the method’s ability to detect defect locations and sizes. The detailed specifications of the specimens are provided below.

- GFRP specimens

**Figure 10:**
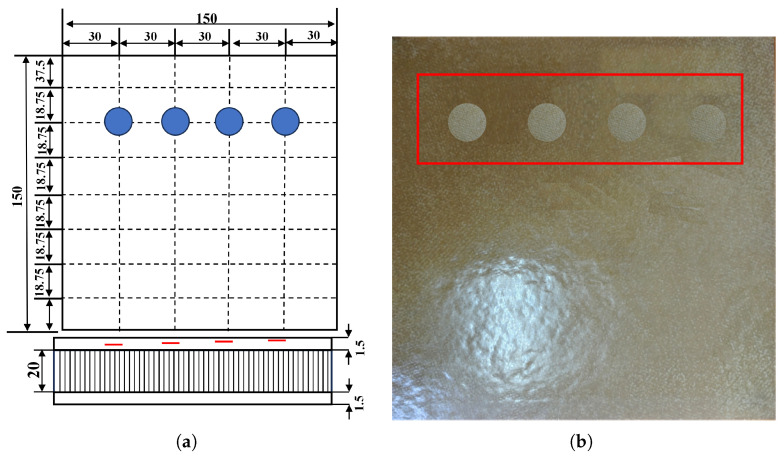
Design and actual image of the GFRP specimen. (**a**) Design diagram (unit:mm). (**b**) Actual photograph. The red box indicates the location of the defect.

The defect design diagram and the actual photograph of the fabricated defect region in the GFRP specimen are presented in Figure 10, respectively. The skins of the specimen were manufactured from a moderate-temperature-curing #18 glass-fiber woven prepreg, with a single-ply thickness of 0.3 mm and a ply orientation of ±45°. Both the upper and lower skins are composed of five plies each. Four simulated defects were designed and fabricated within the specimen. A total of four artificial defects were designed and manufactured in the specimen. These defects were positioned below the first through fourth ply layers, respectively, and each has a diameter of 10 mm. Under each designated ply layer, a PTFE thin film with a diameter of 10 mm and a thickness of 0.02 mm was placed. To verify the ground truth of the simulated PTFE delaminations in the GFRP specimen, the stacking sequence and PTFE insertion positions were strictly controlled during lay-up according to the design figure. After curing, the specimen was inspected using visual inspection of each ply during lay-up, thickness measurement of the cured laminate, and manual cross-checking of the PTFE film locations against the design drawing. Since the PTFE films were placed at known ply interfaces with specified diameters and thickness, the true defect size and depth can be regarded as known and are used as the reference labels in this study.

- QFRP specimens

**Figure 11:**
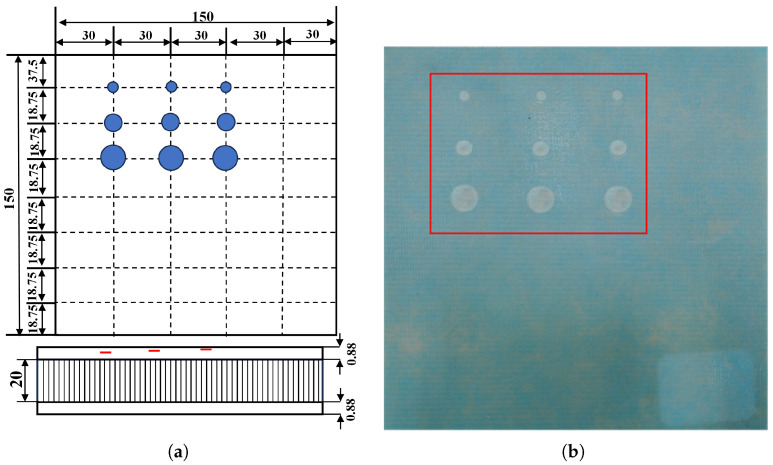
Design and actual image of the QFRP specimen. (**a**) Design diagram (unit:mm). (**b**) Actual photograph. The red box indicate the locations of defects.

As shown in Figure 11, the defect design schematic is illustrated in the left panel, and a photograph of the quartz fiber composite specimen is shown in the right panel. The specimen is constructed in a sandwich configuration, in which the face sheets are made from QW220/3218 epoxy prepreg (Wells Advanced Materials (Shanghai) Co., Ltd., Shanghai, China). Each ply is 0.22 mm thick with fibers oriented at ±45°. Both the upper and lower skins are composed of 4 plies. A J-69B adhesive film (Heilongjiang Institute of Chemical Engineering, Harbin, China) with a thickness of 0.35 mm serves as the bonding layer. The core is a 20 mm thick Nomex honeycomb (Teyi (Shanghai) New Material Co., Ltd., Shanghai, China) with a regular hexagonal cell geometry. A total of nine artificial defects are pre-implanted in the specimen. The defects are sequentially located beneath the first, second, and third plies from the adhesive interface, with one column under each ply. The diameters of the defects in these three columns are 3 mm, 5 mm, and 9 mm, respectively. A PTFE thin film, 0.02 mm thick and with a diameter of 3 mm, 5 mm, or 9 mm matching the designated ply layer, is placed beneath each respective ply. For the QFRP specimen, the PTFE inserts are embedded at the prescribed interfaces. During fabrication, each PTFE film is placed and photographed after lay-up of the corresponding ply, and the final laminate thickness is checked after curing. Therefore, the location and in-plane size of each artificial delamination are known a priori from the manufacturing process and serve as the ground truth for evaluating the detection and classification performance.

#### 3.2.2. Data Collection

In this study, a continuous S-shaped raster scanning strategy was employed for THz-TDS imaging. Time-domain waveforms were acquired point-by-point across the sample surface. The distance between adjacent acquisition points, defined as the scanning step size, directly determines the spatial resolution of the system. During operation, the robotic arm first performed a continuous line scan along the *x*-axis, then stepped a preset distance along the *y*-axis, enabling complete areal coverage. For signal acquisition, a complete time-domain waveform was acquired in approximately 45 ps, with each waveform comprising 1300 temporally sampled data points. This configuration ensured full coverage of the temporal features of the THz pulse.

**Figure 12:**
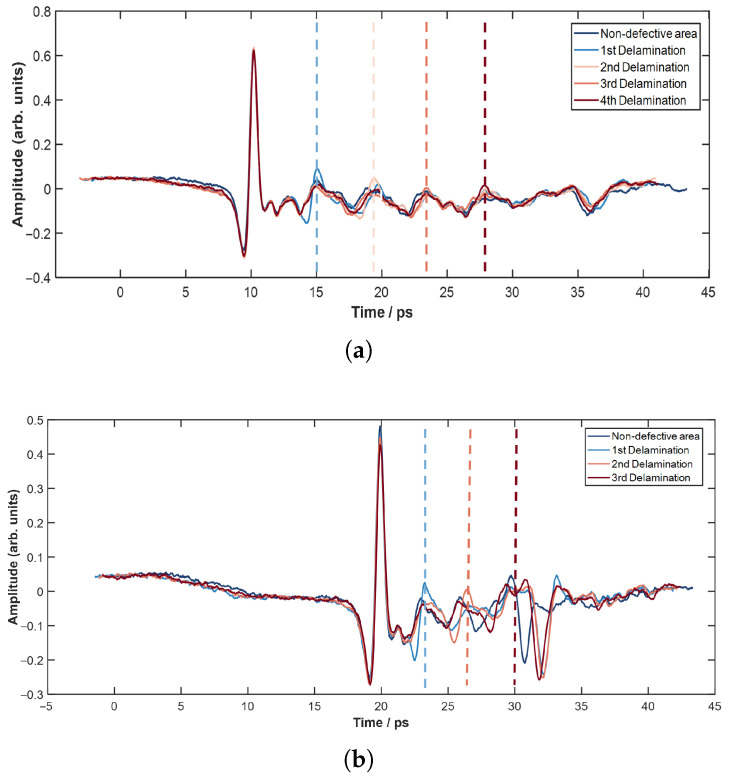
Waveforms of the specimen. (**a**) GFRP waveform. (**b**) QFRP waveform. Dash lines indicate the peaks corresponding to defects.

According to the acquisition strategy described above, a complete terahertz time-domain pulse waveform was captured at each spatial sampling position. Representative raw time-domain waveforms taken from typical areas of the glass fiber and quartz fiber samples are displayed in Figure 12, respectively. In the defect-free regions of both materials, a well-defined primary pulse shape was observed in the signals. By contrast, in the defective regions, clear reflected pulses and noticeable phase shifts were detected. These changes in waveform morphology and characteristics in the time domain provide the foundation for subsequent defect detection and identification.

In this experiment, two separate datasets were prepared: one for GFRP and the other for QFRP. For the GFRP specimen, the scanned area was 130 mm × 50 mm with a step size of 0.5 mm in both the *x* and *y* directions, resulting in 261 × 101 spatial sampling points (26,361 pixels). For the QFRP specimen, the scanned area was 90 mm × 90 mm with the same step size of 0.5 mm, yielding 181 × 181 spatial sampling points (32,761 pixels). The GFRP dataset included 5000 time-domain waveform samples, while the QFRP dataset contained 4000 samples, each comprising 1300 data points. For the purposes of model training and assessment, each dataset was divided into training, validation, and test subsets in a 6:2:2 proportion. The test subset was an entirely independent, unlabeled dataset that remained completely unseen by the model throughout the training phase.

## 4. Results and Discussion

### 4.1. Model Parameters

Time-frequency spectrograms of size 1 × 32 × 32 are fed into local time-frequency and frequency-domain branch. A simplified resnet backbone is employed in each branch for hierarchical feature extraction. The raw time-domain signal with a length of 1300 (L=1300) is taken as the input to time-frequency domain branch. Through above process, the local time-frequency feature map $F_{t}$ and the frequency-domain feature map $F_{f}$ are generated, both having a channel dimension of 16 ($d_{m}=16$). A Transformer encoder coupled with Manifold Mixup is employed to extract fused time-frequency features, resulting in the time-frequency fusion feature $F_{tf}$. The Transformer encoder has two layers, and the number of cycles for Manifold Mixup is four (k=4).

The hyperparameters of our neural network were set as follows: a batch size of 128, training for 100 epochs, and the Adam algorithm was selected as the optimizer. An early stopping strategy was employed, which halted the training process if the validation accuracy showed no improvement over 20 consecutive epochs. The model with the highest validation accuracy during this period was saved for final evaluation. To enhance the model’s generalization ability, a cross-entropy loss function with label smoothing (smoothing factor =0.1) was adopted. All experiments were conducted on a single computer system running Windows 11, equipped with an Intel(R) Core(TM) Ultra 7 265KF processor, 32 GB of RAM, and an NVIDIA GeForce RTX 5070 GPU. The model was developed and tested using the PyTorch 1.7.0 deep learning framework within an Anaconda3 environment.

#### 4.1.1. GFRP Comparative Experiment

**Figure 13:**
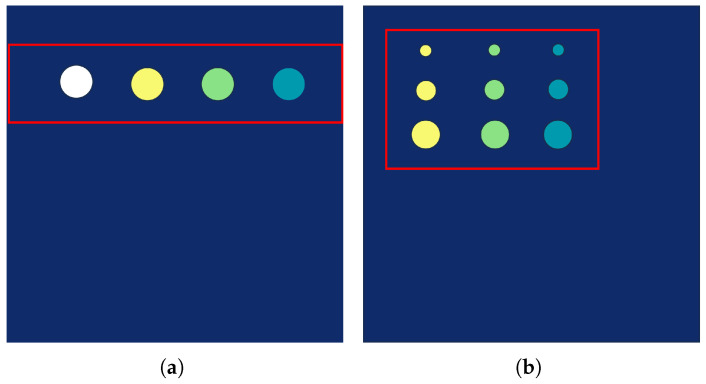
Ideal defect layout in the specimen. (**a**) GFRP. (**b**) QFRP. The red boxes indicate the locations of defects.

To evaluate the effectiveness of the proposed TFFN, it was first compared with Transformer-based neural networks [24], AlexNet [25], ResNet-34 [35], MCLDNN [36], and DACNN [37] on the glass fiber dataset. The comparison was carried out using accuracy, precision, recall, and the average F1-Score as metrics, and the corresponding confusion matrices were generated. In addition, defect visualization images were used to further demonstrate the performance of each method. To reduce the influence of non-defective background regions and enable clearer inspection of the classification results, the subsequent visualizations concentrated on the pre-embedded defect regions, namely the area enclosed by the box in Figure 13.

**Figure 14:**
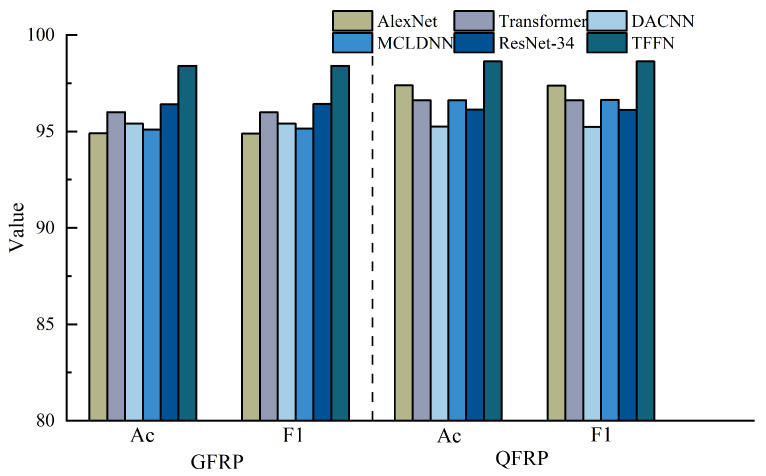
Visualization of comparative experiments on GFRP.

**Figure 15:**
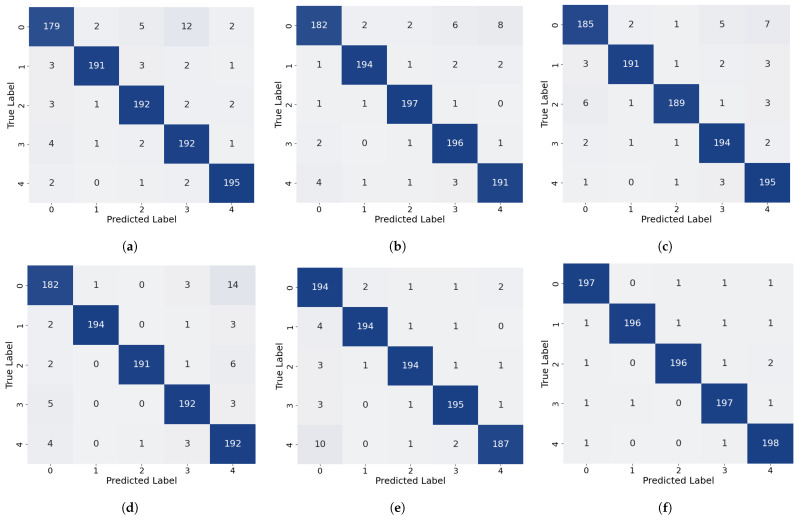
Confusion matrix for the GFRP. (**a**) Alexnet. (**b**) Transformer. (**c**) DACNN. (**d**) MCLDNN. (**e**) Resnet-34. (**f**) TFFN.

As shown in Table 1 and illustrated in Figure 14, the proposed method attains the highest overall accuracy among all compared approaches and delivers superior performance in most categories. Specifically, it achieves the best precision for Label 0, Label 1, and Label 3 (98.01%, 99.49%, and 98.01%, respectively) and the highest recall for Label 0, Label 1, Label 3, and Label 4 (98.50%, 98.00%, 98.50%, and 99.00%, respectively). For the remaining labels, our method’s performance is very close to that of the top-performing models: its precision for Label 2 is only 0.49% lower than that of the best MCLDNN, its precision for Label 4 is just 0.37% lower than that of the best ResNet-34, and its recall for Label 5 is close to that of the best Transformer. These results suggest that our method provides a more uniformly balanced performance across all categories. The confusion matrix in Figure 15 further details the classification outcomes: our approach shows the sharpest and most concentrated main diagonal, reflecting the fewest total misclassifications, whereas all other methods display more pronounced misclassification patterns. The F1-Score combines precision and recall into a single metric, providing a more holistic evaluation of model performance. As shown in Table 1, our method achieves the highest F1-Score of 98.40% among all methods considered. This finding reinforces that our approach preserves high precision while also delivering strong recall, thereby achieving an effective trade-off and a well-balanced overall performance.

**Figure 16:**
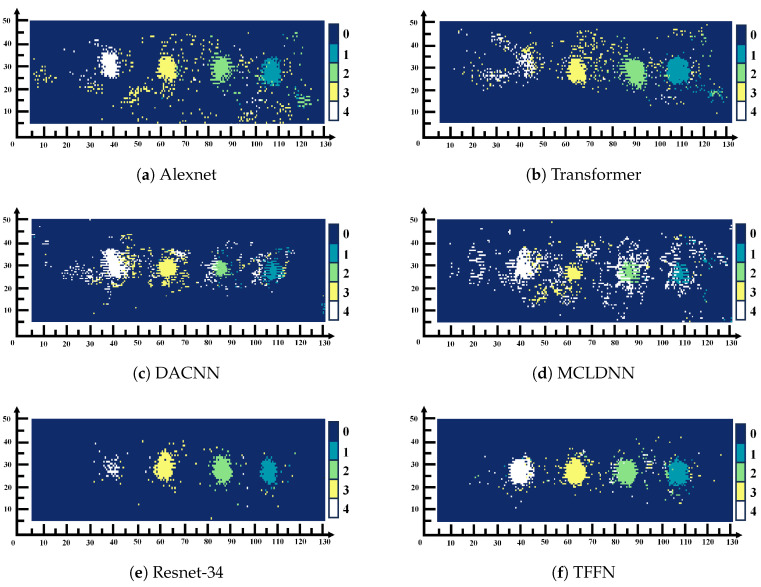
Defect image of the GFRP specimen. The units of both axes are (mm).

To evaluate the generalization ability of TFFN, data from a different composite plate that was excluded from the training process were selected as the test subject. Using label–color mapping, visual images of delamination defects were generated, where Label 0 to Label 4 correspond to normal data and Defect 1 to Defect 4, respectively. As shown in Figure 16, misclassifications occur to varying degrees across different models. Figure 16a–d exhibit relatively severe misidentification: in Figure 16a, the majority of normal samples are misidentified as Defect 3; in Figure 16b, there is a serious problem with normal samples being misidentified as Defect 4 within the Defect 4 region; in Figure 16c,d, the recognition performance for Defects 1–3 is poor, and the identification accuracy in the main defect areas is notably lower than with other methods. In contrast, the recognition performance of Figure 16e,f is significantly better than that of the other methods. Compared with Figure 16e, Figure 16f exhibits a relatively lower recognition rate in the main defect areas, especially for Defect 4, whose recognition rate is markedly lower than that in Figure 16e. In summary, TFFN delivers outstanding performance: it captures the majority of the primary delamination defect regions while keeping the rate of false detections in non-defect areas low.

#### 4.1.2. QFRP Comparative Experiment

The superior performance of the proposed method on glass fiber composites is verified in Section 4.2.1. To further assess its ability to generalize to different composite materials, the method is now applied to QFRP specimens in this section. A systematic evaluation is carried out using the same baseline methods and evaluation metrics as those used for the GFRP dataset.

**Figure 17:**
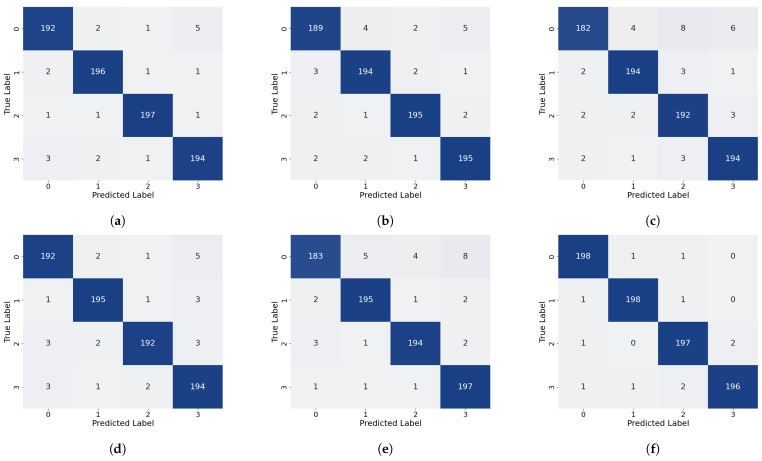
Confusion matrix for the QFRP. (**a**) Alexnet. (**b**) Transformer. (**c**) DACNN. (**d**) MCLDNN. (**e**) Resnet-34. (**f**) TFFN.

As shown in Table 2 and Figure 14, our method also demonstrates strong performance on the quartz dataset: it attains the highest overall accuracy, the highest precision for Label 0, Label 1, and Label 3, and the highest recall for Label 0 and Label 1. For categories where it is not strictly optimal, the difference from the best performing methods is very small: both precision and recall for Label 2 are only about 0.5% lower than the optimal AlexNet, and the recall for Label 3 is just 0.5% lower than the optimal AlexNet. As shown in Figure 17, the main diagonal of our method’s confusion matrix is the sharpest and most concentrated among all methods, mirroring its behavior on the glass fiber dataset. In addition, our method achieves the highest average F1-Score across all methods, confirming its superior overall performance on the quartz dataset.

**Figure 18:**
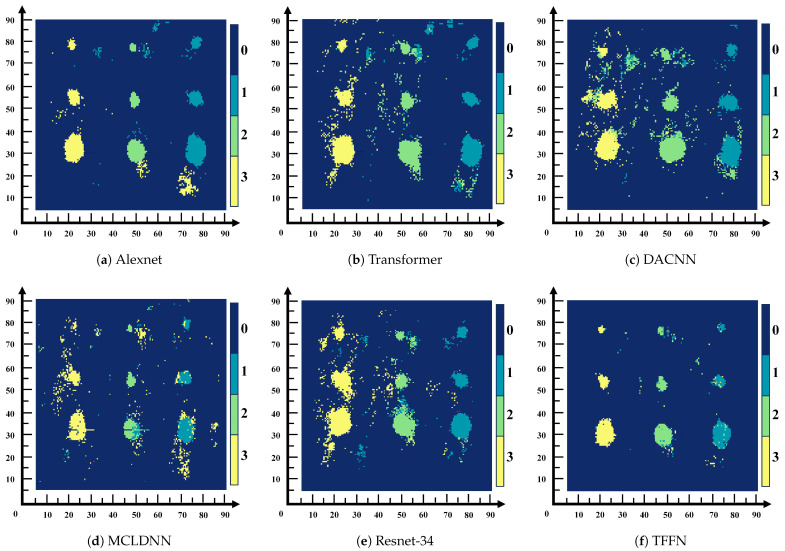
Defect image of the QFRP specimen. The units of both axes are millimeters (mm).

To verify the generalization capability of the proposed approach, data from an untrained composite panel were selected for testing. By applying a label-to-color mapping strategy, a hierarchical visualization of defects was obtained, in which Labels 0–3 corresponded to the normal area and Defects 1–3, respectively. This mapping allowed for a stratified visual representation of the defects, where Label 0 corresponded to the intact area and Labels 1–3 corresponded to Defects 1–3. The visualization results are shown in Figure 18. TFFN (Figure 18f) maintained robust recognition performance on the QFRP dataset, producing images with clear defect boundaries and complete category regions that closely align with the true defect distribution. AlexNet (Figure 18a) likewise yielded generally good recognition outcomes. Nonetheless, relative to AlexNet, TFFN achieved higher recognition accuracy in the local areas around Label 1 and Label 3, with fewer misclassification errors. The other methods display varying levels of recognition deficiencies: Figure 18b substantially misclassifies normal regions as defects and introduces considerable noise near Label 2 and Label 3; Figure 18c performs poorly on deeper defects such as Label 2 and Label 3, leading to extensive missed or erroneous detections; Figure 18e performs satisfactorily on Label 1 but still produces numerous misclassified regions around Label 2 and Label 3. Figure 18d presents clear defect contours but has difficulty distinguishing adjacent defects, with particularly obvious misidentifications near the lowest defect of Label 2. Collectively, these findings highlight the strong generalization capability of the proposed method on the QFRP dataset.

The robust performance of TFFN is attributed to its cross-domain deep feature fusion. Through a three-branch architecture integrating local time-frequency, frequency, and time-frequency domains, local details are preserved in the local time-frequency branch; channel–spatial–frequency attention is introduced in the frequency branch to enhance damage-sensitive frequency bands; a Transformer encoder and Manifold are utilized in the time-frequency fusion branch to obtain fused features across domains; and multi-branch information is finally integrated via cross-branch attention mechanisms. This design not only enhances the robustness of defect recognition but also reduces misclassification in non-defect regions through cross-domain cross-validation, resulting in superior generalization performance. In the case of other comparative methods, either complementary time-frequency domain information is not fully integrated during feature extraction or simplistic fusion strategies lacking deep cross-domain feature integration mechanisms are employed, leading to limited generalization capabilities in complex defect recognition scenarios.

**Figure 19:**
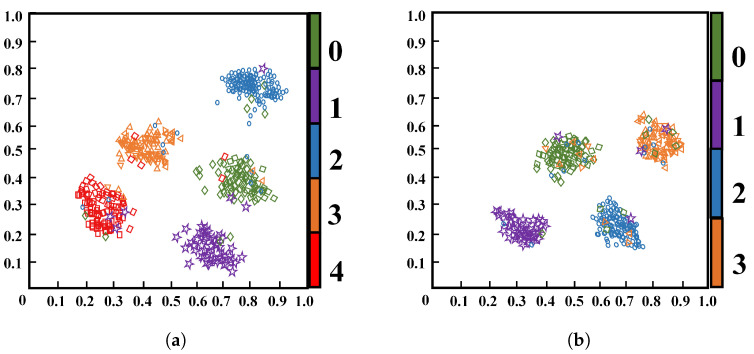
T-SNE visualization of the features learned by TFFN. (**a**) GFRP. (**b**) QFRP.

The effectiveness of the proposed cross-domain deep fusion mechanism is intuitively verified in the feature space. Figure 19 shows the t-SNE visualization of the features extracted by TFFN from the GFRP and QFRP datasets. The horizontal and vertical axes correspond to the first and second t-SNE embedding dimensions, respectively. The coordinate values are linearly rescaled to the range [0, 1] for visualization and are unitless; they do not represent the physical size of the sample. As shown, distinct and well-separated clusters emerge for each defect class in both datasets, characterized by small intra-class distances and large inter-class separations. This indicates that the features learned by TFFN via cross-domain fusion exhibit strong discriminative capability among different categories, thereby supporting its outstanding classification accuracy and generalization performance.

### 4.2. Ablation Experiments

To validate the design rationale of the proposed three-branch network (TFFN) architecture, the frequency-domain enhancement module (CSFANet), and the cross-branch attention fusion mechanism, ablation studies were conducted on both the GFRP and QFRP datasets. The experiments were performed at two granularities: branch-level and module-level ablation. For branch-level ablation, the local time-frequency branch (TFFN_d-ltf_), the frequency-domain branch (TFFN_d-f_), and the time-frequency fusion branch (TFFN_d-tf_) were individually removed. For module-level ablation, the CSFANet module was removed (TFFN_d-csfa_), and the cross-branch attention fusion mechanism was replaced with simple concatenation (TFFN_sim_) and linear fusion (TFFN_linear_), respectively, to evaluate the contribution of each component. In these ablation studies, accuracy, precision, recall, and the macro-averaged F1-Score were adopted as quantitative metrics to assess the importance of each branch and module design. Additionally, confusion matrices were used for visual analysis to complement the quantitative evaluation.

#### 4.2.1. Branch Ablation Experiment

**Figure 20:**
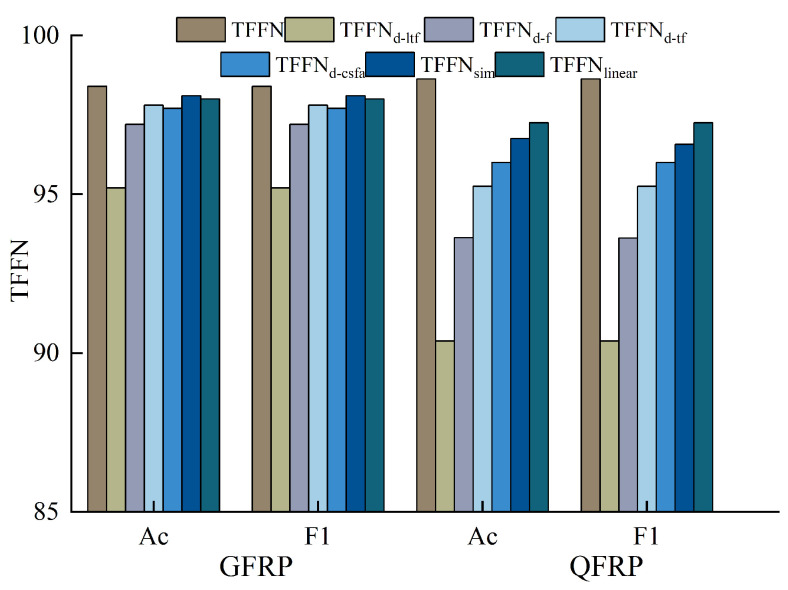
Performance comparison of the ablation experiments.

**Figure 21:**
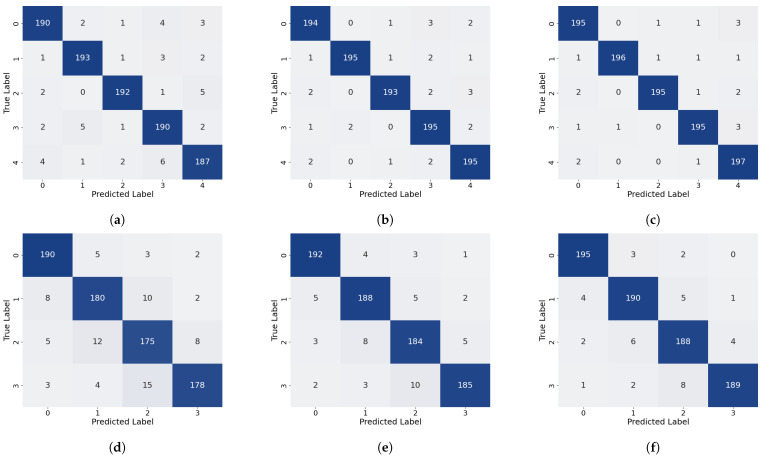
Confusion matrix for the branch experiment. (**a**) GFRP-TFFN_d-ltf_. (**b**) GFRP-TFFN_d-f_. (**c**) GFRP-TFFN_d-tf_. (**d**) QFRP-TFFN_d-t_. (**e**) QFRP-TFFN_d-f_. (**f**) QFRP-TFFN_d-tf_.

To validate the rationality of the branch design, a branch-level ablation study is presented in this subsection. A comparison of performance across three scenarios, each representing the removal of a different branch, is presented in Table 3 and Figure 20 and Figure 21.

The removal of the local time-frequency branch (TFFN_ltf_) led to a marked degradation in defect detection performance across both datasets. For the GFRP dataset, the overall accuracy dropped by 3.4%, and the precision for deep defects (Label 3 and Label 4) fell by 4.87% and 3.57%, respectively, accompanied by a simultaneous reduction in recall. This demonstrates that, without local time-frequency features, the model’s ability to distinguish deep defects is severely weakened. In the QFRP dataset, the effect was even more substantial: overall accuracy declined by 8.25%, and precision, recall, and F1-Score all decreased markedly. The most pronounced performance losses were observed for defect classes Label 1 and Label 2. Analysis of the confusion matrix showed a large number of missed detections and incorrect classifications for these two defect categories. Performance degradation was also observed on both datasets after removing the frequency-domain branch (TFFN_f_), though the specific patterns differed. For the GFRP dataset, the overall accuracy dropped by 1.2%, and the precision for Label 3 decreased from 98.01% to 95.59%. This suggests that frequency-domain features play a distinct role in boosting the accuracy of deep defect recognition. In the QFRP dataset, eliminating the frequency-domain branch led to a 5% reduction in accuracy, with Label 1 and Label 2 exhibiting the most pronounced performance declines. This reflects how crucial the frequency-domain branch is for extracting spectral features when identifying interlayer defects in quartz materials. A relatively small but consistent effect was observed when the time–frequency fusion branch (TFFN_tf_) was removed. For the GFRP dataset, the overall accuracy declined by just 0.6%, but the precision for Label 4 decreased by 1.91%, indicating that time–frequency domain information still offers additional classification support for deeper defects. In the QFRP dataset, accuracy decreased by 3.38%, with Label 1 and Label 2 again being the most impacted classes. This further confirms that the time–frequency domain branch contributes complementary features, particularly for mid-layer defects.

The rationality of the three-branch architectural design is validated by the observations above. Its effectiveness stems from the targeted modeling of the physical interaction between terahertz signals and defects: the local time-frequency branch captures pulse delay and morphological changes, enabling precise depth localization and detailed characterization of defects; the frequency-domain branch focuses on the spectral composition of the signal, exhibiting high sensitivity to material discrimination and boundary extraction of mid-to-surface defects; the time-frequency fusion branch enhances feature robustness in complex scenarios through joint representation. By systematically integrating the features from all three branches, effective identification of defects across different materials and varying depths is achieved.

#### 4.2.2. Module Ablation Experiment

**Figure 22:**
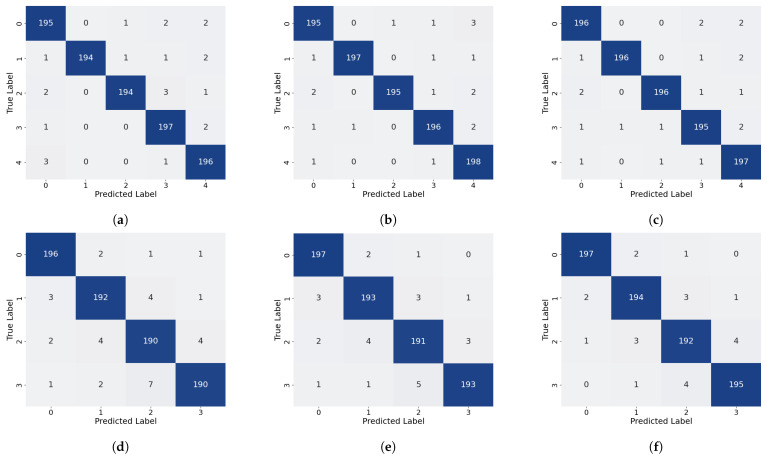
Confusion matrix for the ablation experiment. (**a**) GFRP-TFFN_d-csfa_. (**b**) GFRP-TFFN_sim_. (**c**) GFRP-TFFN_linear_. (**d**) QFRP-TFFN_d-csfa_. (**e**) QFRP-TFFN_sim_. (**f**) QFRP-TFFN_linear_.

A module-level ablation study is conducted in this subsection to validate the design effectiveness of CSFANet and the cross-branch attention fusion mechanism. The performance differences among three configurations are compared: (1) removing CSFANet, (2) replacing the cross-branch attention with simple concatenation, and (3) replacing the cross-branch attention with linear fusion [38]. The quantitative results are reported in Table 4, while the corresponding visual results are illustrated in Figure 20 and Figure 22. In addition, several fusion strategies within the time–frequency branch, including concatenation, linear fusion, cross-attention, and Manifold Mixup, were compared with assess their impact on the overall fusion performance.

To validate the necessity of the CSFANet module, ablation experiments were conducted by removing the module. On the GFRP dataset, the impact of excluding CSFANet was relatively small: overall accuracy declined by 0.7%. The precision for Label 0 and Labels 2–4 fell by 1–2%. Interestingly, without CSFANet, the precision for Label 1 increased to 100%, but this was accompanied by a 0.5% decrease in recall. This result does not indicate a flaw in the CSFANet design; instead, it demonstrates that CSFANet serves to balance precision and recall, leading to more consistent detection performance across labels. On the QFRP dataset, removing CSFANet led to a 2.63% reduction in accuracy, along with concurrent declines in precision, recall, and F1-Score. The most notable effects were observed for shallow defects (Label 1) and medium-depth defects (Label 2), where precision dropped by 3.00% and 3.95%, respectively. This indicates that CSFANet significantly improves the classification of shallow and medium-depth defects. Overall, these findings show that CSFANet strengthens defect feature representation and is critical for accurately classifying shallow and medium-depth defects in quartz materials.

To validate the advantages of the proposed cross-branch attention mechanism, experiments were conducted comparing it against alternative fusion strategies: simple concatenation (TFFN_sim_) and linear fusion (TFFN_linear_). On the GFRP dataset, replacing attention with simple concatenation led to only a 0.30% reduction in both accuracy and average F1-Score, indicating minimal surface-level impact. In contrast, the precision for deep defects (Label 4) declined by 2.28%, highlighting that the attention mechanism is crucial for effectively fusing features associated with deep defects. When using linear fusion, the negative effect was slightly stronger: accuracy and average F1-Score decreased by 0.40%, and the precision for deep defects (Label 4) dropped by 0.97%. From these results, it is further confirmed that complex defect characteristics are better captured by the attention mechanism than by linear fusion. The contribution of the attention mechanism is even more evident on the QFRP dataset. A 1.87% decline in accuracy, along with reductions in key metrics including precision, was observed as a result of simple concatenation. In particular, the precision for shallow defects (Label 1) fell by 2.50%, and that for medium-depth defects (Label 2) decreased by 2.51%. This indicates that the attention mechanism substantially improves the discriminative performance for shallow and medium-depth defects. Similarly, the use of linear fusion resulted in a clear drop in performance, lowering both accuracy and the average F1-Score by 1.38%. The precision of Labels 1–3 declined to different degrees, whereas the normal class (Label 0) was not impacted. These findings provide additional evidence for the importance of the cross-branch attention mechanism in achieving effective multi-branch feature fusion.

The effectiveness of the CSFANet module and the cross-branch attention mechanism is confirmed by the results above. A three-dimensional attention mechanism is utilized by the CSFANet module to accurately emphasize defect-sensitive spectra and refine feature representations, thereby improving the model’s capability to detect defects in composite materials. In comparison, straightforward concatenation and linear fusion yield only static or linear combinations of features and cannot dynamically modulate the contribution of each branch according to the spatiotemporal-frequency characteristics of the input signal. By contrast, inter-branch correlations are computed by the cross-branch attention mechanism to adaptively highlight the feature dimensions most pertinent to the current defect, thus strengthening the model’s classification robustness and generalization performance under complex conditions.

In addition, we conducted further ablation experiments on the time-frequency branch to evaluate the impact of different fusion methods on model performance. We tested four strategies: simple concatenation (Concat), linear fusion (Linear), cross-attention (CA), and the Manifold Mixup method proposed in this paper. The experimental results are shown in Table 5. The results show that Concat and Linear achieved accuracy rates of 97.80% and 98.10%, respectively, on the GFRP dataset, and 97.25% and 97.50% on the QFRP dataset. This indicates that even with the most basic static fusion method, there remains a certain degree of complementarity between temporal and frequency domain features. In contrast, CA achieved lower accuracy (97.70% for GFRP and 97.12% for QFRP), while the Manifold Mixup method proposed in this paper achieved the best results on both datasets. The reason for these performance differences lies in the fact that the time-domain and frequency-domain features of terahertz signals differ in their physical properties and statistical structures; they are not naturally aligned. Concat and Linear methods only perform static combinations and cannot address distribution biases between cross-domain features, making it difficult to achieve higher fusion quality. Although CA possesses dynamic alignment capabilities, it requires learning complex matching mappings for cross-domain features and introduces additional parameters. With limited data scale, this can easily lead to unstable attention distributions and further exacerbate the risk of overfitting. In contrast, Manifold Mixup performs continuous interpolation of cross-domain features in the latent space, making the fusion process more consistent in terms of distribution and thereby effectively mitigating the instability caused by cross-domain differences.

The effectiveness of the proposed model was quantitatively demonstrated via ablation experiments: removing any individual branch caused a clear drop in performance, thereby verifying both the utility and the complementary roles of the three-branch architecture. In addition, the CSFANet module strengthens feature discriminability while the cross-branch attention mechanism enhances feature fusion, with both contributing distinct and complementary performance gains. Furthermore, a comparative analysis of fusion strategies indicates that Manifold Mixup offers a more stable and generalizable method for fusing time-frequency features.

### 4.3. Impact of Frequency-Band Weight Initialization

**Figure 23:**
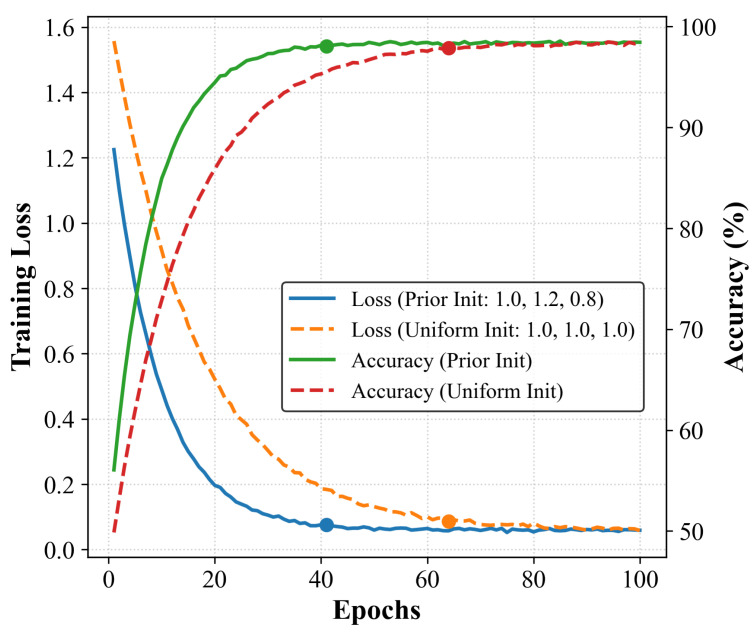
Comparison of loss and accuracy convergence under different weight initialization strategies.

To evaluate the impact of different band weight initialization methods on model training and performance, this study compared two strategies: non-uniform initialization based on prior knowledge and standard uniform initialization. As shown in Figure 23, there are significant differences in the convergence curves of the loss function and accuracy between the two strategies; the dots in the figure mark the time points at which each strategy entered the stable convergence phase.

The results show that prior initialization provides a more effective optimization direction in the early stages of training, allowing the model to reach a stable plateau after approximately 42 epochs; in contrast, under uniform initialization, the loss and accuracy curves do not begin to level off until around the 63rd epoch. Although there is a difference in convergence speed between the two, in the later stages of training, both initialization methods converge stably to nearly identical performance levels, with virtually the same final classification accuracy. Since band weights are learnable parameters, they are automatically adjusted during backpropagation to the ratio that best matches the task; even in the absence of prior differences, the model can gradually learn a band importance distribution similar to that of the prior initialization. Therefore, the advantage of prior-based initialization lies primarily in accelerating early convergence, rather than determining the final performance.

In summary, the proposed band-weighted strategy exhibits good robustness during initialization. Both the prior-based and uniform initialization methods ultimately converge to comparable classification performance, further demonstrating the stability and reliability of this method in practical applications.

### 4.4. Computational Efficiency Analysis

To comprehensively evaluate the computational overhead of TFFN, we conducted standardized inference performance tests on an NVIDIA GeForce RTX 5070 GPU (batch size = 1) and compared the results with baseline models. To ensure fairness, the input dimensions of all baseline models were standardized to 1 × 32 × 32 or THz time-domain sequences of length 1300, thereby avoiding any bias in inference efficiency caused by input dimensions. A quantitative evaluation of the model’s overall operational efficiency was conducted in this study, measuring three key metrics: the number of trainable parameters, the average inference time per THz signal, and the resulting frames per second (FPS). These metrics were selected to comprehensively reflect the model’s performance in terms of computational overhead and real-time capabilities.

As shown in Table 6, the experimental results reveal significant differences among the models in terms of the number of parameters, inference efficiency, and classification performance. TFFN has 9.887 million parameters. Although this is higher than DACNN (0.097 million), AlexNet (2.47 million), and ResNet 34 (0.46 million), it still falls within the category of lightweight networks, and the model size remains within deployable limits. Meanwhile, TFFN’s average inference time is 9.30 ms, corresponding to a frame rate of 107.5 FPS. Although its architecture includes three feature extraction branches, a cross-domain fusion module, and a sequence modeling unit—resulting in higher overall computational complexity than other lightweight models—its inference speed remains above 100 frames per second, meeting the real-time requirements of composite material non-destructive testing tasks.

In terms of inference efficiency, AlexNet is the fastest (3.17 ms, 316.0 FPS), but its average classification accuracy is only 96.14%, significantly lower than that of TFFN; ResNet-34 has an inference time of 5.750 ms (173.9 FPS), with an average accuracy of 96.26%, which is also lower than TFFN. MCLDNN and Transformer have inference times of 6.20 ms and 7.10 ms, respectively, with frame rates slightly higher than TFFN; however, their average accuracy remains in the range of 95.86%–96.31%, making it difficult to effectively distinguish between multiple defect classes with subtle differences.

A comprehensive comparison reveals that while TFFN is slightly slower in inference speed than some shallow or single-branch models, its average accuracy across the two datasets reaches 98.52%, significantly outperforming all other baseline models. Through three-branch heterogeneous feature extraction, cross-domain interaction and fusion, and temporal modeling mechanisms, TFFN is able to more fully capture the complementary information of THz TDS signals in the time, frequency, and time-frequency domains, thereby significantly improving classification robustness and recognition accuracy. Consequently, while achieving millisecond-level inference speeds, TFFN demonstrates a more pronounced advantage in accuracy, successfully balancing precision with real-time performance, making it more suitable for engineering-oriented non-destructive testing scenarios.

### 4.5. Analysis of the Characteristics of Complex Permittivity Variations and Detection Sensitivity

**Figure 24:**
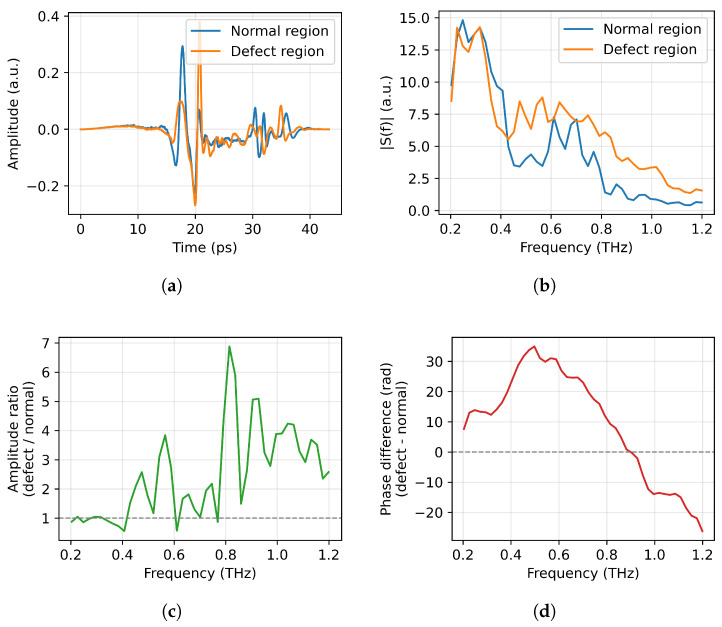
Time–frequency analysis of terahertz responses in normal and defect regions. (**a**) Time-domain waveforms. (**b**) Frequency-domain amplitude spectra. (**c**) Amplitude ratio between defect and normal regions. (**d**) Phase difference as a function of frequency.

The propagation behavior of terahertz waves in composite materials is directly determined by their complex permittivity:

(30) $$\varepsilon^{*}\left(f\right)=\varepsilon^{\prime }\left(f\right)-j\varepsilon^{\prime \prime }\left(f\right)$$

where $\varepsilon^{\prime }\left(f\right)$ primarily influences the propagation speed of electromagnetic waves, phase delay, and interface reflection characteristics, while $\varepsilon^{\prime \prime }\left(f\right)$ reflects absorption loss and spectral energy attenuation. Since laminated composites consist of a multiphase structure comprising fibers, resin, adhesive layers, and honeycomb cores, intrinsic dispersion and significant spatial inhomogeneities coexist within them. When delamination defects form, air gaps ($\varepsilon^{\prime }\approx 1$) replace the resin layers ($\varepsilon^{\prime }\approx$ 2.5–3.0), leading to a decrease in local polarization capacity, changes in the interface structure, and an increase in scattering paths. Consequently, the equivalent complex permittivity undergoes systematic shifts in its real and imaginary parts, as well as in its frequency-dependent behavior. Due to the structural complexity, this study employs the equivalent complex permittivity $\varepsilon_{\mathrm{eff}}^{*}\left(f\right)$ as the analytical parameter to explain the differences in terahertz response between the normal and defect regions. To visually demonstrate this effect at the signal level, we analyze QFRP signals; the results are shown in Figure 24.

In the real part, the defect region exhibits a lower effective permittivity due to the increased proportion of air, causing the overall value of $\varepsilon_{\mathrm{eff}}^{\prime }\left(f\right)$ to decrease. At the same time, since air exhibits almost no dispersion, the slope of $\varepsilon_{\mathrm{eff}}^{\prime }\left(f\right)$ as a function of frequency in the defect region is weaker than that in the normal region. This effect can be directly observed in the time-domain waveform shown in Figure 24a: the peak position of the main pulse in the defect region shifts forward, and the shapes of the rising and falling edges are broadened, reflecting the group delay difference caused by the change in propagation phase velocity. Furthermore, the echo intensity and arrival time in the defect region differ from those in the normal region, further indicating that the phase accumulation difference caused by the change in refractive index is amplified during multi-interface propagation. The phase difference spectrum in Figure 24d provides more direct evidence: the defect region exhibits a continuous phase shift relative to the normal region across the entire frequency band, with a larger shift rate in the mid- and upper-mid-frequency bands. This indicates that the dispersion laws of the phase velocity in the two regions are markedly different, representing a typical macroscopic manifestation of the variation in $\varepsilon_{\mathrm{eff}}^{\prime }\left(f\right)$.

In terms of the imaginary part, the layered defect simultaneously reduces intrinsic loss, enhances interfacial loss, and alters the scattering path, resulting in distinct directional and frequency-band characteristics in the variation of $\varepsilon_{\mathrm{eff}}^{\prime }\left(f\right)$. The amplitude spectrum in Figure 24b shows that the low-frequency region of the defect is nearly identical to that of the normal region, indicating that their effective losses are close at low frequencies, i.e., $\varepsilon_{\mathrm{eff},\;\mathrm{defect}}^{\prime }\left(f\right)\approx \varepsilon_{\mathrm{eff},\;\mathrm{normal}}^{\prime }\left(f\right)$. In the mid-frequency range, however, the amplitude spectrum begins to exhibit an overall shift and spectral distortion. This reflects the fact that the original polarization loss peak of the resin matrix is attenuated by the air gap, while the newly introduced interfacial reflection causes energy to be amplified at certain frequencies and attenuated at others. The amplitude ratio plot in Figure 24c reveals this frequency band structure more clearly: In the mid-frequency range, an alternating pattern of “positive peak–negative trough–positive peak” appears, indicating that $\varepsilon_{\mathrm{eff},\;\mathrm{defect}}^{\prime }\left(f\right)$ exhibits a dispersion perturbation in this frequency region characterized by “first greater than normal, then less than normal, and then greater than normal,” reflecting the redistribution of the equivalent imaginary part under the combined effects of weakened absorption loss and enhanced interface reflection. In the high-frequency band, the defect spectrum decays more rapidly, with an amplitude-to-normal ratio significantly below one, indicating enhanced scattering loss that causes high-frequency energy to decay more quickly, corresponding to $\varepsilon_{\mathrm{eff},\;\mathrm{defect}}^{\prime }\left(f\right)>\varepsilon_{\mathrm{eff},\;\mathrm{normal}}^{\prime }\left(f\right)$. This three-segment pattern of “low-frequency approximation, mid-frequency fluctuation, and high-frequency enhanced attenuation” directly reflects the direction and magnitude of the change in the imaginary part of the complex permittivity.

In summary, layered defects cause an overall decrease in the real part of the equivalent permittivity and frequency-band-dependent perturbations in the imaginary part while altering the frequency-dependent behavior of both components. This results in a series of distinguishable differences in amplitude, phase, and energy structures of terahertz signals in the time domain, frequency domain, and local time-frequency domain. The features in Figure 24 collectively constitute the intuitive manifestation of the aforementioned changes in the complex permittivity at the macroscopic signal level. Based on this, the TFFN proposed in this study can simultaneously capture phase perturbations, spectral distortion, and energy migration—multiscale information resulting from variations in $\varepsilon_{\mathrm{eff}}^{\prime }\left(f\right)$ and $\varepsilon_{\mathrm{eff}}^{\prime \prime }\left(f\right)$—within a joint time-frequency neighborhood. Furthermore, the model’s attention automatically focuses on the mid-frequency sensitive regions where differences are most pronounced in Figure 24, indicating that the key features extracted by the network align with the variation patterns of the dielectric constants. Ultimately, accuracy rates of 98.40% and 98.63% were achieved on the GFRP and QFRP datasets, respectively, demonstrating that the model maintains a highly sensitive response to complex permittivity perturbations, thereby exhibiting excellent cross-material applicability and overall detection sensitivity.

### 4.6. Noise Robustness Evaluation

THz time-domain signals acquired in the laboratory typically exhibit a high signal-to-noise ratio and minimal external interference. However, in practical engineering applications, the detection environment is often affected by factors such as equipment vibration, electromagnetic noise, structural coupling, and fluctuations in ambient temperature, causing the signals to contain varying degrees of random noise. Therefore, it is necessary to construct test data under different noise conditions based on the normal samples to evaluate the model’s stability and adaptability in non-ideal environments. To this end, we generate three test sets with typical noise intensities by adding zero-mean Gaussian white noise to the original signals, without altering the structure or labels of the original samples. The signal-to-noise ratios are set to 20 dB, 10 dB, and 0 dB, respectively. Signals under different noise levels are input into each model to compare how their classification accuracy and F1 scores change as noise increases, thereby analyzing the models’ noise robustness. The results are shown in the Table 7.

As can be seen from the table, the performance of all models declines as noise intensity increases, though the rate of decline varies. Traditional CNN architectures (AlexNet, DACNN) exhibit a more pronounced drop in accuracy when noise is increased. Transformer models perform relatively stably under mild and moderate noise but exhibit some degradation under strong noise (0 dB). In contrast, TFFN maintains the highest accuracy and F1 scores under all noise conditions, retaining an accuracy of over 97% even under strong noise (0 dB), significantly outperforming other models. The results show that TFFN maintains stable classification performance across different noise levels, demonstrating strong adaptability to varying environmental conditions. To evaluate the effectiveness of the method described in this paper in real-world scenarios, the following section presents performance tests conducted on multi-layer composite structures.

### 4.7. Robustness Analysis Under Equivalent Multi-Layer Conditions

In practical aerospace and industrial applications, composite components often consist of 20 or even 30 layers or more. However, it is challenging to fabricate ultra-multilayer composite specimens with internal defects. To comprehensively evaluate the performance of the TFFN model under conditions such as increased layer count and environmental interference, we employ a stress testing method based on a physical degradation model, which is widely recognized in the field of NDT. The input for this method is derived from the GFRP and QFRP composite material datasets constructed in this paper, with an SNR of 40 dB in the raw signals. We use real signals that the model has not previously encountered as input and construct an ultra-multilayer degradation test set by applying mathematical operators that follow the physical laws governing terahertz wave propagation in thick media. Subsequently, these degraded signals are directly fed into the pre-trained TFFN model to evaluate its performance under equivalent ultra-multi-layer conditions.

#### 4.7.1. Construction of the Terahertz Signal Degradation Model

To ensure the validity and accuracy of the simulated signals, the degradation model developed in this study strictly adheres to the three core physical mechanisms governing the propagation of terahertz waves in multilayer media, and the key parameters are rigorously calibrated against reference standards:

- Beer-Lambert absorption and Rayleigh scattering

The attenuation of terahertz waves in multilayer GFRP/QFRP does not scale uniformly across the entire frequency range. According to the theory of electromagnetic wave propagation, the resin matrix produces a fixed background absorption, while the interwoven fiber network within the composite causes intense Rayleigh scattering. Therefore, we apply a frequency-dependent attenuation filter to the spectrum X(f) of the input test signal x(t):

(31) $$X_{att}\left(f\right)=X\left(f\right)\cdot \exp\left(-\left(\alpha_{0}+\alpha_{1}f^{2}\right)\cdot d_{eq}\right)$$

where $d_{eq}$ is the simulated equivalent thickness. In this study, the background absorption coefficient is strictly calibrated as $\alpha_{0}=0.2\;{\mathrm{mm}}^{-1}$, and the coefficient representing the severe loss due to high-frequency Rayleigh scattering is calibrated as $\alpha_{1}=0.8$ mm^−1^/THz^2^. This physical process causes the signal energy to decrease exponentially and results in severe high-frequency cutoff.

- Multiple interface reflection superposition

As the number of layers increases, minute impedance mismatches between layers cause the forward-propagating waveform to be superimposed with countless faint reflections. This study introduces a physically meaningful delayed reflection term in the time domain:

(32) $$x_{mr}\left(t\right)=F^{-1}\left[X_{att}\left(f\right)\right]+\sum_{i=1}^{k}\gamma_{i}\cdot x\left(t-\Delta t_{i}\right)$$

where $\gamma_{i}$ represents the interlayer weak reflection coefficient and $\Delta t_{i}$ represents the time-of-flight delay corresponding to the interlayer thickness. Physically, this term reproduces the typical waveform distortion and tail observed at the trailing edge of the main pulse in ultramultilayer materials.

- SNR degradation

In real-world multi-layer non-destructive testing, the energy of defect echoes at deep interfaces typically decays rapidly, causing the SNR of the final received signal to approach or even fall below 0 dB. To address this, Gaussian white noise n(t) is injected into the signal to simulate real-world industrial conditions.

(33) $$x_{degraded}\left(t\right)=x_{mr}\left(t\right)+n\left(t\right),\;SNR=10\log_{10}\left(\frac{P_{signal}}{P_{noise}}\right)$$

where $x_{\mathrm{degraded}}\left(t\right)$ is the degraded observed signal, $x_{mr}\left(t\right)$ is the valid echo signal obtained by considering only multiple reflections, and n(t) denotes zero-mean Gaussian white noise; $P_{\mathrm{signal}}$ and $P_{\mathrm{noise}}$ denote the average powers of the valid signal and noise, respectively, within a given sampling window.

#### 4.7.2. Model Validation

**Figure 25:**
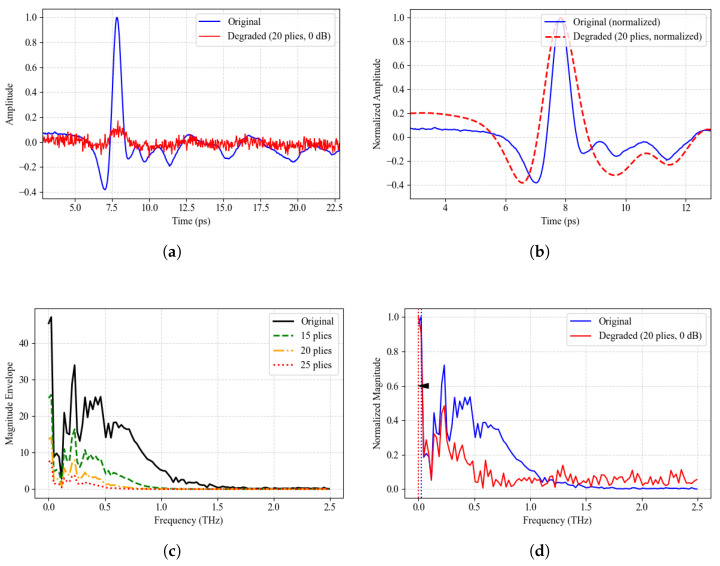
Time–Frequency domain validation of the terahertz signal degradation model. (**a**) Time-domain comparison between original and 20-ply degraded signals. (**b**) Peak-aligned, normalized time-domain pulses showing pulse broadening after degradation. (**c**) Spectrum envelope evolution with increasing equivalent ply number (0, 15, 20, 25 plies). (**d**) frequency-domain comparison between original and 20-ply degraded signals.

To verify whether the constructed degradation model accurately reflects the propagation degradation mechanism of terahertz waves in ultra-multilayer composite materials, we constructs equivalent 10-, 20-, and 25-layer degradation signals based on the raw terahertz signal from a specific measurement point on the QFRP and conduct a comparative analysis of the signal evolution characteristics in both the time and frequency domains. In this degradation model, the layer number denotes an equivalent propagation thickness rather than the actual ply thickness. Each equivalent layer is set to 0.2 mm to conveniently model cumulative attenuation. Thus, 15, 20, and 25 layers correspond to equivalent propagation distances of 3.0 mm, 4.0 mm, and 5.0 mm, respectively. Figure 25 demonstrates spectral shape, and time-domain pulse morphology.

Figure 25a compares the normalized original shallow-penetration terahertz echo with the degraded signal under conditions equivalent to 20 layers and 0 dB. In the original signal, the main pulse exhibits a high-amplitude, well-defined peak with low background noise; in contrast, in the degraded signal, the amplitude of the main peak is significantly reduced, and the waveform is largely overwhelmed by high-level noise. According to model calculations, the amplitude of the main peak in the 20-layer equivalent signal is attenuated to approximately 15.1% of that in the original shallow-layer signal. This phenomenon is consistent with empirical observations in the non-destructive testing of ultra-multilayer composites. In terms of time-domain morphology, the loss of high-frequency components not only causes a decrease in amplitude but also alters the pulse width and the steepness of the edges. To eliminate the influence of amplitude differences, the original signal and the equivalent 20-layer degraded signal were normalized separately and compared by aligning their main peaks. The results are shown in Figure 25b. The degraded main pulse exhibits typical time-domain broadening compared with the original main pulse. The mid-to-high-frequency components in the original signal help maintain the sharp edges of the time-domain waveform, but these are attenuated during propagation through the ultra-multilayer coating, resulting in the broadening of the main pulse along the time axis.

Figure 25c shows the amplitude envelope of the degraded signal for the original signal and for equivalent layer counts of 15, 20, and 25. Within the main energy distribution band, the spectral amplitude decreases as the equivalent layer count increases, indicating that the degradation model introduces energy attenuation that accumulates with thickness. As the number of layers increases, the high-frequency end of the spectrum is gradually suppressed and approaches noise. This characteristic is consistent with the Beer–Lambert-type transmission law of terahertz waves in thick composite materials and the physical mechanism whereby high-frequency components are more easily absorbed and scattered. To provide a more intuitive comparison of changes in spectral shape, Figure 25d shows a normalized superposition of the original signal and the spectrum of a signal equivalent to a 20-layer, 0 dB degraded signal. It can be seen that within the frequency bands where the main energy is concentrated, the amplitude of the degraded spectrum is lower than that of the original spectrum, and the spectral components on the high-frequency side are more easily attenuated. Beyond approximately 1.0 THz, the amplitudes of both curves approach zero; however, due to noise and normalized perturbations, the effective bandwidth of the degraded signal narrows significantly, which is consistent with physical reality.

#### 4.7.3. Experimental Results and Analysis

To generate terahertz time-domain waveform data with varying degrees of degradation, we map the original test set to three typical degradation scenarios by adjusting the equivalent propagation thickness and injecting noise. Specifically, condition a ($c_{a}$) corresponds to an equivalent propagation thickness of 15 layers and a SNR of approximately 20 dB; condition b ($c_{b}$) corresponds to an equivalent coverage thickness of 20 layers and a SNR of approximately 10 dB; condition c ($c_{c}$) corresponds to an equivalent coverage thickness of 25 layers and a SNR of approximately 0 dB. These conditions are used to simulate varying degrees of signal attenuation and noise interference. The results are shown in Table 8.

As the equivalent coverage thickness increased from 15 layers to 25 layers and the SNR decreased from approximately 20 dB to approximately 0 dB, both the $A_{C}$ and F1 scores of all models showed a downward trend, with the performance decline becoming more pronounced as the degradation worsened. This trend holds consistent across both the GFRP and QFRP datasets, indicating that the constructed degradation models can reasonably reflect physical laws such as the energy attenuation of terahertz waves in multilayer media. Although overall performance declined as degradation intensified, TFFN consistently outperformed the comparison models under all conditions and demonstrated greater robustness: under the most severe condition c, it still maintained accuracy rates of 97.32% and 98.13%, whereas the comparison methods experienced a significant drop, indicating that conventional architectures are susceptible to feature blurring and noise interference under strongly degraded THz waveforms. Under operating $c_{c}$, TFFN still maintains relatively stable recognition performance, which is attributed to its internal feature modeling approach. On the one hand, traditional networks rely heavily on the absolute amplitude and sharp edges of time-domain waveforms; however, these features are degraded during propagation due to amplitude attenuation, pulse broadening, and significant loss of high-frequency components. In contrast, TFFN uses CSFANet to adaptively focus on the mid-frequency energy structure, which is less affected by thickness-induced attenuation, and employs a Transformer to extract more stable spatiotemporal joint features. On the other hand, the cross-branch attention mechanism within the network automatically adjusts branch weights under low signal-to-noise ratio conditions, thereby suppressing background noise at the feature level and highlighting structural information related to defects. Overall, this design—which simultaneously utilizes multi-view features and adaptively adjusts branch contributions—enables TFFN to demonstrate superior robustness against deep-layer degradation effects such as pulse broadening, amplitude attenuation, and high-frequency component loss. Consequently, it maintains a high recognition accuracy close to that under normal operating conditions even under operating condition c.

### 4.8. Frequency Domain Attention Visualization and Analysis

To further verify that the CSFANet module can indeed focus on damage-sensitive frequency bands during frequency-domain feature extraction, a visual analysis of frequency-domain attention comprising spectral attention distributions and subband attention matrices is supplemented. Based on the frequency-domain energy distribution characteristics of terahertz time-domain signals, the 0–2.5 THz band is divided into three sub-bands: the low-frequency band (0–0.3 THz), the mid-frequency band (0.3–1.0 THz), and the high-frequency band (1.0–2.5 THz). Among these, the mid-frequency band is the key frequency range most strongly correlated with layered defects. To further refine the attention distribution within this critical frequency range, this study builds upon the aforementioned three-segment division by subdividing the 0–2.5 THz band into seven sub-bands, thereby enabling a clearer representation of the network’s differentiated attention patterns within the mid- frequency band.

**Figure 26:**
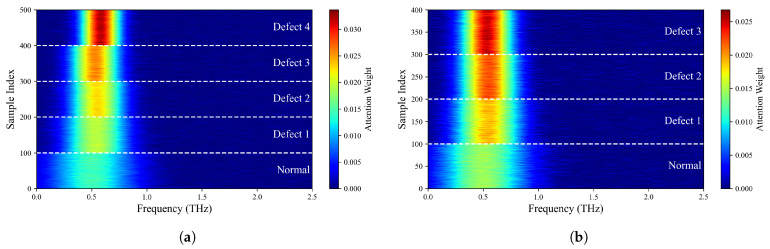
Frequency-domain attention heatmap. (**a**) GFRP. (**b**) QFRP.

Representative samples were selected from each class in the GFRP and QFRP datasets, and the frequency-domain attention heatmap is shown in Figure 26. The results show that CSFANet’s high attention is primarily concentrated in the mid-frequency band of the three-band division. The 0.4–0.7 THz band is the core attention peak, which naturally extends to the 0.7–1.0 THz band. This attention distribution indicates that CSFANet can actively focus on the physically sensitive frequency bands associated with the mechanisms of layered defects. Specifically, the model maintains a consistently high level of attention in the 0.1–1.0 THz range, which covers the characteristic spectral responses of layered interfaces caused by effects such as dielectric constant discontinuities and enhanced interfacial reflection. Furthermore, the attention peak formed by the network near 0.4–0.7 THz aligns with the enhanced absorption and dispersion changes commonly observed in GFRP/QFRP materials within this frequency range, reflecting the model’s ability to actively identify key frequency information that is more sensitive to layered damage. This result indicates that CSFANet does not focus on random noise points or training biases, but rather on frequency bands associated with the material’s physical mechanisms, thereby validating the model’s reliability and interpretability in frequency-domain feature extraction.

**Figure 27:**
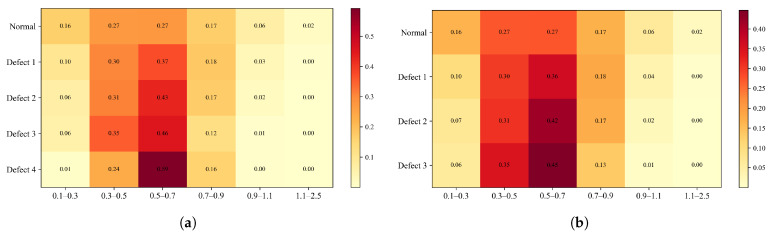
Frequency-domain attention matrix. (**a**) GFRP. (**b**) QFRP.

Furthermore, the frequency-domain attention matrix based on a division into seven fixed sub-bands is shown in Figure 27. The results show that the highest attention distribution for all defect categories is concentrated in the three sub-bands of 0.3–0.9 THz, further validating the critical role of the mid-frequency band in hierarchical defect detection.

The distribution patterns described above clearly demonstrate that the key frequency bands targeted by the model are not generated randomly, but rather have a clear physical correspondence with changes in dielectric properties caused by delamination defects in composite materials. In summary, by employing a dual perspective of spectral attention heatmaps and subband energy matrices, we demonstrate that the CSFANet module can adaptively focus on damage-sensitive frequency bands with clear physical significance, thereby significantly enhancing the interpretability and reliability of frequency-domain feature extraction. Compared with the defect category, the attention distribution of normal samples is more dispersed across all frequency bands, whereas defect samples exhibit a slightly more concentrated response in the mid-frequency range, indicating that the presence of interlayer discontinuities introduces relatively consistent spectral perturbations in this region.

## 5. Conclusions

A composite material delamination defect identification network is proposed in this paper, with its core methodology based on three-branch feature extraction and fusion. By integrating local time-frequency, frequency domain, and time-frequency-domain features, the limitations of single-domain representations are overcome. In the local time-frequency branch, a lightweight ResNet is employed to extract pulse-shape features. In the frequency branch, the CSFANet module is introduced on top of the temporal features, where deformable convolution and channel-spatial-frequency band attention are utilized to achieve dynamic frequency band decomposition and feature enhancement. The time-frequency branch is equipped with Manifold Mixup to enable deep cross-domain feature fusion. Furthermore, a cross-branch attention fusion mechanism is designed to dynamically weight features from different branches, thereby promoting information complementarity and enhancing discriminability among heterogeneous features. The superior performance of the proposed method is demonstrated through comparative experiments on two self-built datasets, while the effectiveness of each module is further verified by ablation studies. Therefore, an accurate and robust intelligent detection approach for composite delamination defects is provided by the presented TFFN, which exhibits strong practical value and application potential.

Although the proposed method has demonstrated high accuracy under controlled laboratory conditions, several practical factors must still be considered in real-world industrial applications. Surface moisture strongly absorbs and scatters terahertz waves, thereby altering local dielectric properties and reducing the effective signal-to-noise ratio; in such cases, the model may need to be retrained using moisture-calibrated data or undergo domain adaptation. Furthermore, high-speed industrial scanning typically employs larger step sizes and fewer averaging passes, which further reduces the signal-to-noise ratio and spatial resolution. For online inspection, the proposed framework can be combined with terahertz sources operating at higher repetition rates and sparse/linear array scanning, thereby maintaining reliable defect characterization capabilities under high-throughput conditions. We will investigate this further in future work.

## Figures and Tables

**Table 1:** Comparative experiment on the GFRP dataset.

Label	AlexNet				Transformer				DACNN			
	P	R	F1	$A_{c}$	P	R	F1	$A_{c}$	P	R	F1	$A_{c}$
0	93.72	89.50	94.89	94.90	95.79	91.00	95.99	96.00	93.91	92.50	95.40	95.40
1	97.95	95.50			97.98	97.00			97.95	95.50		
2	95.48	96.00			97.52	98.50			97.93	94.50		
3	91.43	96.00			94.23	98.00			94.63	97.00		
4	97.01	97.50			94.55	95.50			92.86	97.50		
Label	MCLDNN				ResNet-34				TFFN			
	P	R	F1	$A_{c}$	P	R	F1	$A_{c}$	P	R	F1	$A_{c}$
0	93.33	91.00	95.14	95.10	90.65	97.00	96.42	96.40	98.01	98.50	98.40	98.40
1	99.49	97.00			98.48	97.00			99.49	98.00		
2	99.48	95.50			97.98	97.00			98.99	98.00		
3	96.00	96.00			97.50	97.50			98.01	98.50		
4	88.07	96.00			97.91	93.50			97.54	99.00		

Values with underline indicate the best performance in each metric. The unit of the data is percentage (%).

**Table 2:** Comparative experiment on the QFRP dataset.

Label	AlexNet				Transformer				DACNN			
	P	R	F1	$A_{c}$	P	R	F1	$A_{c}$	P	R	F1	$A_{c}$
0	96.97	96.00	97.37	97.38	96.43	94.50	96.62	96.62	96.81	91.00	95.24	95.25
1	97.51	98.00			96.52	97.00			96.04	97.00		
2	98.50	98.50			97.50	97.50			93.20	96.00		
3	96.52	97.00			96.06	97.50			95.10	97.00		
Label	MCLDNN				ResNet-34				TFFN			
	P	R	F1	$A_{c}$	P	R	F1	$A_{c}$	P	R	F1	$A_{c}$
0	96.48	96.00	96.63	96.62	96.83	91.50	96.11	96.12	98.51	99.00	98.63	98.63
1	97.50	97.50			96.53	97.50			99.00	99.00		
2	97.96	96.00			97.00	97.00			98.01	98.50		
3	94.63	97.00			94.62	98.50			98.99	98.00		

Values with underline indicate the best performance in each metric. The unit of the data is percentage (%).

**Table 3:** Model performance of the branch ablation experiment.

Label	GFRP-TFFN_d-ltf_				GFRP-TFFN_d-f_				GFRP-TFFN_d-tf_			
	P	R	F1	$A_{c}$	P	R	F1	$A_{c}$	P	R	F1	$A_{c}$
0	95.48	95.00	95.20	95.20	97.00	97.00	97.20	97.20	97.01	97.50	97.80	97.80
1	96.02	96.50			98.98	97.00			99.49	98.00		
2	97.46	96.00			98.47	96.50			98.98	97.50		
3	93.14	95.00			95.59	97.50			97.99	97.50		
4	93.97	93.50			96.06	97.50			95.63	98.50		
Label	QFRP-TFFN_d-ltf_				QFRP-TFFN_d-f_				QFRP-TFFN_d-tf_			
	P	R	F1	$A_{c}$	P	R	F1	$A_{c}$	P	R	F1	$A_{c}$
0	92.23	95.00	95.38	95.38	95.05	96.00	93.63	93.63	96.53	97.50	95.25	95.25
1	89.55	90.00			92.61	94.00			94.53	95.00		
2	86.21	87.50			91.09	92.00			92.61	94.00		
3	93.68	89.00			95.85	92.50			97.42	94.50		

The unit of the data is percentage (%).

**Table 4:** Model performance of the module ablation experiment.

Label	GFRP-TFFN_d-csfa_				GFRP-TFFN_sim_				GFRP-TFFN_linear_			
	P	R	F1	$A_{c}$	P	R	F1	$A_{c}$	P	R	F1	$A_{c}$
0	96.53	97.50	97.70	97.70	97.50	97.50	98.10	98.10	97.51	98.00	98.00	98.00
1	100.00	97.49			99.49	98.50			99.49	98.00		
2	98.98	97.00			99.49	97.50			98.98	98.00		
3	96.57	98.50			98.00	98.00			97.50	97.50		
4	96.55	98.00			96.12	99.00			96.57	98.50		
Label	QFRP-TFFN_d-csfa_				QFRP-TFFN_sim_				QFRP-TFFN_linear_			
	P	R	F1	$A_{c}$	P	R	F1	$A_{c}$	P	R	F1	$A_{c}$
0	97.03	98.00	96.00	96.00	97.04	98.50	96.75	96.75	96.53	97.50	97.25	97.25
1	96.00	98.00			96.50	96.50			97.00	97.00		
2	94.06	95.00			95.50	95.50			96.00	96.00		
3	96.94	95.00			97.97	96.50			97.50	97.50		

The unit of the data is percentage (%).

**Table 5:** Model performance of the fusion strategy ablation experiment.

Strategy	GFRP Dataset				QFRP Dataset				Params (M)
	**P**	**R**	**F1**	$A_{c}$	**P**	**R**	**F1**	$A_{c}$	
Concat	97.80	97.83	97.80	97.80	97.28	97.25	97.26	97.25	9.887
Linear	98.13	98.10	98.10	98.10	97.52	97.50	97.50	97.50	9.891
CA	97.73	97.70	97.70	97.70	97.13	97.12	97.13	97.12	10.242
Ours	98.40	98.40	98.40	98.40	98.63	98.63	98.63	98.63	9.887

The unit of the data is percentage (%).

**Table 6:** Comprehensive comparison of model efficiency and classification performance.

Model	Params (M)	FLOPs (M)	Time (ms)	FPS	Accuracy (%)		
					**GFRP**	**QFRP**	**Mean**
DACNN	0.097	0.14	2.40	416.7	95.40	95.25	95.33
AlexNet	2.471	14.81	3.17	316.0	94.90	97.38	96.14
ResNet-34	0.462	10.92	5.75	173.9	96.40	96.11	96.26
MCLDNN	4.216	12.41	6.20	161.3	95.10	96.62	95.86
Transformer	0.801	18.20	7.10	140.8	96.00	96.62	96.31
TFFN	9.887	32.83	9.30	107.5	98.40	98.63	98.52

All accuracy values are percentages (%).

**Table 7:** Performance comparison under different noise levels.

GFRP	AlexNet		Transformer		DACNN		MCLDNN		ResNet-34		TFFN	
	$A_{C}$	F1	$A_{C}$	F1	$A_{C}$	F1	$A_{C}$	F1	$A_{C}$	F1	$A_{C}$	F1
Normal	94.90	94.89	96.00	95.99	95.40	95.40	95.10	95.14	96.40	96.42	98.40	98.40
20 dB	93.80	93.82	95.80	95.81	95.22	95.21	94.90	94.92	95.98	95.97	98.36	98.38
10 dB	92.41	92.42	95.41	95.42	94.72	94.71	94.31	94.32	95.01	95.00	98.12	98.11
0 dB	89.61	89.62	93.67	93.65	94.01	94.02	93.87	93.88	93.80	93.81	97.71	97.72
QFRP	AlexNet		Transformer		DACNN		MCLDNN		ResNet-34		TFFN	
	$A_{C}$	F1	$A_{C}$	F1	$A_{C}$	F1	$A_{C}$	F1	$A_{C}$	F1	$A_{C}$	F1
Normal	97.37	97.38	96.62	96.62	95.25	95.24	96.62	96.63	96.12	96.11	98.63	98.63
20 dB	96.87	96.86	96.51	96.52	95.11	95.12	96.40	96.41	95.87	95.88	98.62	98.61
10 dB	94.62	94.61	95.97	95.99	94.74	94.72	95.97	95.98	94.81	94.80	98.42	98.40
0 dB	92.24	92.23	93.41	93.42	93.31	93.33	94.01	94.02	92.41	92.40	98.18	98.19

All values are given in percentage (%).

**Table 8:** Model performance comparison on GFRP and QFRP datasets under different degradation conditions.

GFRP	AlexNet		Transformer		DACNN		MCLDNN		ResNet-34		TFFN	
	$A_{C}$	F1	$A_{C}$	F1	$A_{C}$	F1	$A_{C}$	F1	$A_{C}$	F1	$A_{C}$	F1
Normal	94.90	94.89	96.00	95.99	95.40	95.40	95.10	95.14	96.40	96.42	98.40	98.40
$c_{a}$	93.80	93.82	95.60	95.62	95.10	95.12	94.80	94.82	95.40	95.40	98.32	98.31
$c_{b}$	92.41	92.42	94.81	94.82	94.62	94.61	94.11	94.12	93.11	93.10	97.90	97.91
$c_{c}$	89.61	89.62	92.71	92.72	93.92	93.91	92.90	92.91	89.80	89.81	97.32	97.31
QFRP	AlexNet		Transformer		DACNN		MCLDNN		ResNet-34		TFFN	
	$A_{C}$	F1	$A_{C}$	F1	$A_{C}$	F1	$A_{C}$	F1	$A_{C}$	F1	$A_{C}$	F1
Normal	97.37	97.38	96.62	96.62	95.25	95.24	96.62	96.63	96.12	96.11	98.63	98.63
$c_{a}$	96.01	96.05	96.41	96.42	95.01	95.00	96.31	96.30	95.04	95.02	98.59	98.61
$c_{b}$	93.51	93.53	95.69	95.70	94.21	94.20	95.80	95.81	93.01	93.00	98.42	98.41
$c_{c}$	91.04	91.03	92.31	92.30	93.01	93.11	93.61	93.60	89.50	89.51	98.13	98.11

All values are given in percentage (%).

## Data Availability

All data are contained within the article.

## References

[1] Liu Z., Kong X., Cai C., Peng H., Zhang J. (2024). Internal defect characterization of bridge cables based on Terahertz time-domain spectroscopy and deep learning. Eng. Struct..

[2] Jung S.H., Yeo W.H., Maeng I., Ji Y., Oh S.J., Ryu H.C. (2025). Self-supervised deep-learning for efficient denoising of terahertz images measured with THz-TDS system. Expert Syst. Appl..

[3] Hu J., Liu W., Mao X., Li W., Chen J., Xie F. (2025). Research on rubber inclusion defect detection based on terahertz time-domain spectroscopy technology and image fusion. NDT E Int..

[4] Azad M.M., Kumar P., Kim H.S. (2024). Delamination detection in CFRP laminates using deep transfer learning with limited experimental data. J. Mater. Res. Technol..

[5] Xiao W., Sha G., Zuo H., Cao M., Radzieński M., Kudela P., Ostachowicz W. (2025). Deep learning-aided guided wavefield imaging of delaminations in composite laminates. Mech. Syst. Signal Process..

[6] Gupta R., Mitchell D., Blanche J., Harper S., Tang W., Pancholi K., Baines L., Bucknall D.G., Flynn D. (2021). A review of sensing technologies for non-destructive evaluation of structural composite materials. J. Compos. Sci..

[7] Shetu M. (2024). A Review of Nondestructive Testing Methods for Aerospace Composite Materials. J. Comput. Mech. Manag.

[8] Jamil J., Yusup E.M., Osman S.A. (2024). Non-destructive testing (NDT) method for defect detection in glass fibre reinforced plastic/polymer (GFRP/GRP) composite materials structures: A review. J. Adv. Res. Micro Nano Eng..

[9] Xie L., Lian Y., Du F., Wang Y., Lu Z. (2024). Optical methods of laser ultrasonic testing technology in the industrial and engineering applications: A review. Opt. Laser Technol..

[10] Wu J.H., Yang J.S., Zhang X.Y., Fu L.L., Li S., Wu L.Z., Schmidt R., Schröder K.U. (2025). A neural network-based air-coupled ultrasonic damage detection method for composite honeycomb sandwich structure. Mech. Syst. Signal Process..

[11] Yagdjian H., Gurka M. (2025). One-dimensional N-layer thermal modelling for effective machine learning training data generation for nondestructive testing of composite parts with infrared thermography. Compos. Part B Eng..

[12] Amraei J., Katunin A., Dragan K., Colombo C., Świderski W., Salerno A., Manes A. (2025). Quantification and fusion of thermography and ultrasonic non-destructive testing results for composites with low-velocity impact damage. Compos. Part B Eng..

[13] Zhu P., Wang R., Sivagurunathan K., Sfarra S., Sarasini F., Ibarra-Castanedo C., Maldague X., Zhang H., Mandelis A. (2025). Frequency multiplexed photothermal correlation tomography for non-destructive evaluation of manufactured materials. Int. J. Extrem. Manuf..

[14] ur Rahman M.S., Abou-Khousa M.A., Akbar M.F. (2024). A review on microwave non-destructive testing (NDT) of composites. Eng. Sci. Technol. Int. J..

[15] Li Z., Fei F., Meng Z. (2025). Dielectric-loaded waveguide probe for microwave non-destructive evaluation of glass fibre-reinforced polymer composites. Compos. Struct..

[16] Shi Y., Tong C., Li Y., Zhang K., Liu C., Zhao D., Qiao M., Li X., Cheng H. (2025). Non-destructive evaluation of manufacturing defects in GFRP using cross-correlation method and enhanced terahertz imaging. Sci. China Technol. Sci..

[17] Jang H.L., Lee S.H., Kang L.H. (2025). Non-destructive testing for thickness measurement of the adhesive layer and defect detection in ceramic-metal bonded structures using THz waves. Struct. Health Monit..

[18] Xiao B., Wang Y., Zuo X., Liu L., Jiang W., Zhang Y., Qin J., Zhang D., Xiao L. (2025). Ultra-highly sensitive THz metamaterial sensor based on electromagnetically induced transparency-like effect. Opt. Eng..

[19] Xiao B., Wang Y., Zuo X., Liu L., Qin J., Jiang W., Zhang Y., Xiao L. (2025). Dual-band terahertz metasurface biosensor based on electromagnetically induced transparency effects for antibiotic detection. Opt. Lasers Eng..

[20] Chen X., Zhang Y., Cai G., Zhuo J., Lai K., Ye L. (2022). All-dielectric metasurfaces with high Q-factor Fano resonances enabling multi-scenario sensing. Nanophotonics.

[21] Zhu P., Wei Z., Sfarra S., Usamentiaga R., Steenackers G., Mandelis A., Maldague X., Zhang H. (2025). THz-Super-Resolution Generative Adversarial Network: Deep-Learning-Based Super-Resolution Imaging Using Terahertz Time-Domain Spectroscopy. IEEE Trans. Ind. Inform..

[22] Zhu P., Zhang H., Santulli C., Sfarra S., Usamentiaga R., Vavilov V.P., Maldague X. (2024). Contactless and nondestructive evaluation of residual stress distribution in modified and pure HDPE materials using a novel terahertz method and line-scan thermographic technique. Compos. Part Appl. Sci. Manuf..

[23] Ryu C.H., Park S.H., Kim D.H., Jhang K.Y., Kim H.S. (2016). Nondestructive evaluation of hidden multi-delamination in a glass-fiber-reinforced plastic composite using terahertz spectroscopy. Compos. Struct..

[24] Liu Q., Wang Q., Guo J., Liu W., Xia R., Yu J., Wang X. (2024). A Transformer-based neural network for automatic delamination characterization of quartz fiber-reinforced polymer curved structure using improved THz-TDS. Compos. Struct..

[25] Krizhevsky A., Sutskever I., Hinton G.E. (2017). ImageNet classification with deep convolutional neural networks. Commun. ACM.

[26] Xu Y., Lian G., Zhou H., Hou Y., Zhang H., Zhang L., Yan R., Chen X. (2023). Terahertz transfer characterization for composite delamination under variable conditions based on deep adversarial domain adaptation. Compos. Sci. Technol..

[27] Shen Q., Xiao B., Mi H., Yu J., Xiao L. (2025). Adaptive Learning Filters–Embedded Vision Transformer for Pixel-Level Segmentation of Low-Light Concrete Cracks. J. Perform. Constr. Facil..

[28] Guo J., Yang Y., Li H., Dai L., Huang B. (2024). A parallel deep neural network for intelligent fault diagnosis of drilling pumps. Eng. Appl. Artif. Intell..

[29] Wang J., Xu T., Zhang L., Chang T., Zhang J., Yan S., Cui H.L. (2022). Nondestructive damage evaluation of composites based on terahertz and X-ray image fusion. NDT E Int..

[30] Zhang D., Li L., Zhang J., Ren J., Gu J., Li L., Jiang B., Zhang S. (2024). Quantitative detection of defects in multi-layer lightweight composite structures using THz-TDS based on a U-Net-BiLSTM network. Materials.

[31] Tu W., Zhong S., Zhang Q., Huang Y., Luo M. (2025). Multiple damage identification of epoxy coating structures based on terahertz pulse imaging technology and machine learning. Nondestruct. Test. Eval..

[32] Zhang J.Y., Yang X.K., Ren J.J., Li L.J., Zhang D.D., Gu J., Xiong W.-H. (2024). Terahertz recognition of composite material interfaces based on ResNet-BiLSTM. Measurement.

[33] Xiong W., Ren J., Zhang J., Zhang D., Gu J., Xue J., Chen Q., Li L. (2023). Defect identification in adhesive structures using multi-Feature fusion convolutional neural network. Front. Phys..

[34] Liu Z., Wu Y., Wang K., Man R., He C., Wu B. (2023). Research on Defect Detection Imaging of Ceramic Matrix Composites Based on Terahertz Time-Domain Spectroscopy Technology. J. Mech. Eng..

[35] He K., Zhang X., Ren S., Sun J. Deep residual learning for image recognition. Proceedings of the IEEE Conference on Computer Vision and Pattern Recognition.

[36] Xu J., Luo C., Parr G., Luo Y. (2020). A spatiotemporal multi-channel learning framework for automatic modulation recognition. IEEE Wirel. Commun. Lett..

[37] Huang J., Dwivedi K., Roig G. Deep anchored convolutional neural networks. Proceedings of the IEEE/CVF Conference on Computer Vision and Pattern Recognition Workshops.

[38] Zhang Z., Wang C., Gan C., Sun S., Wang M. (2019). Automatic modulation classification using convolutional neural network with features fusion of SPWVD and BJD. IEEE Trans. Signal Inf. Process. Over Netw..

